# The Genomic Landscape of the Ewing Sarcoma Family of Tumors Reveals Recurrent *STAG2* Mutation

**DOI:** 10.1371/journal.pgen.1004475

**Published:** 2014-07-10

**Authors:** Andrew S. Brohl, David A. Solomon, Wendy Chang, Jianjun Wang, Young Song, Sivasish Sindiri, Rajesh Patidar, Laura Hurd, Li Chen, Jack F. Shern, Hongling Liao, Xinyu Wen, Julia Gerard, Jung-Sik Kim, Jose Antonio Lopez Guerrero, Isidro Machado, Daniel H. Wai, Piero Picci, Timothy Triche, Andrew E. Horvai, Markku Miettinen, Jun S. Wei, Daniel Catchpool, Antonio Llombart-Bosch, Todd Waldman, Javed Khan

**Affiliations:** 1 Oncogenomics Section, Pediatric Oncology Branch, Center for Cancer Research, National Cancer Institute, National Institutes of Health, Bethesda, Maryland, United States of America; 2 Department of Genetics and Genomic Sciences, Icahn School of Medicine at Mount Sinai, New York, New York, United States of America; 3 Department of Pathology, University of California, San Francisco, San Francisco, California, United States of America; 4 The Tumour Bank, The Children's Cancer Research Unit, The Children's Hospital at Westmead, Westmead, New South Wales, Australia; 5 Department of Pathology, University of Valencia, Valencia, Spain; 6 Center for Personalized Medicine, Children's Hospital Los Angeles, University of Southern California Los Angeles, Los Angeles, California, United States of America; 7 Laboratory of Experimental Oncology, Rizzoli Institute, Bologna, Italy; 8 Laboratory of Pathology, Center for Cancer Research, National Cancer Institute, Bethesda, Maryland, United States of America; 9 Department of Oncology, Lombardi Comprehensive Cancer Center, Georgetown University School of Medicine, Washington, District of Columbia, United States of America; University of Washington, United States of America

## Abstract

The Ewing sarcoma family of tumors (EFT) is a group of highly malignant small round blue cell tumors occurring in children and young adults. We report here the largest genomic survey to date of 101 EFT (65 tumors and 36 cell lines). Using a combination of whole genome sequencing and targeted sequencing approaches, we discover that EFT has a very low mutational burden (0.15 mutations/Mb) but frequent deleterious mutations in the cohesin complex subunit *STAG2* (21.5% tumors, 44.4% cell lines), homozygous deletion of *CDKN2A* (13.8% and 50%) and mutations of *TP53* (6.2% and 71.9%). We additionally note an increased prevalence of the *BRCA2* K3326X polymorphism in EFT patient samples (7.3%) compared to population data (OR 7.1, p = 0.006). Using whole transcriptome sequencing, we find that 11% of tumors pathologically diagnosed as EFT lack a typical EWSR1 fusion oncogene and that these tumors do not have a characteristic Ewing sarcoma gene expression signature. We identify samples harboring novel fusion genes including *FUS-NCATc2* and *CIC-FOXO4* that may represent distinct small round blue cell tumor variants. In an independent EFT tissue microarray cohort, we show that STAG2 loss as detected by immunohistochemistry may be associated with more advanced disease (p = 0.15) and a modest decrease in overall survival (p = 0.10). These results significantly advance our understanding of the genomic and molecular underpinnings of Ewing sarcoma and provide a foundation towards further efforts to improve diagnosis, prognosis, and precision therapeutics testing.

## Author Summary

The Ewing sarcoma family of tumors is a group of aggressive cancers that primarily affects the pediatric and young adult population. Increasingly, genomics are being used to better define the disease biology and to identify targets for therapy in many cancer types. Here, we report one of the first and largest genomic studies to date in the Ewing sarcoma family of tumors. Using a combination of modern sequencing techniques in >100 samples, we discover that Ewing sarcomas have a genome that is less complex compared to most cancer types previously surveyed. We find that this cancer is frequently affected by mutations in *STAG2*, a gene that has recently gained attention due to its importance in the biology of several cancer types. We show that Ewing sarcoma patients whose tumors are affected by STAG2 loss may have a worse prognosis. Additionally, we identify a subset of tumors that were diagnosed as Ewing sarcoma that appear to be distinct from the majority based on genetic and molecular characteristics. Our findings help to define the genetic landscape of Ewing sarcoma and provide a starting point for improving individualization of diagnosis, prognosis and treatment in this cancer.

## Introduction

The Ewing sarcoma family of tumors (EFT) is a group of malignant small round blue cell tumors that arise in bone or soft tissue. Ewing sarcoma (ES) is the second most common type of primary bone tumor to affect children and adolescents and accounts for 2.9% of all childhood cancers [1]. Despite advances in multidisciplinary treatment leading to improved outcomes over time for localized disease, long term survival remains poor for patients with metastatic or relapsed disease [2], [3]. The pathological diagnosis of Ewing sarcoma is based on the finding of a small round blue cell tumor (SRBCT) that stains for MIC2 (CD99) but has absence of markers that characterize the other pathologically defined SRBCT variants. In larger centers an EWSR1 break-apart probe is used to detect a fusion event involving this gene, but in most cases this test is not required for a diagnosis of Ewing sarcoma. In previous case series, most EFT cases express one of several reciprocal translocations, most commonly t(11;22)(q24;q12) between the amino terminus of the *EWSR1* gene and the carboxy terminus of the *FLI1* gene found in 85–90% of cases [4], [5]. A number of variant translocations between an alternate member of the *TET* family of RNA-binding proteins and/or an alternate member of the *ETS* family of transcription factors have also been described [6]. Additional structural chromosomal changes are frequently found in EFT, including gain of chromosome 1q, 2, 8, and 12, and losses of 9p and 16q [7]–[9]. Recurrent mutations in known tumor suppressor genes have also been described, though with lower frequency. Most notably, homozygous deletions of *CDKN2A* (which encodes p16^INK4a^) have been detected in 10 to 30 percent of cases and *TP53* mutations in 3 to 14 percent of cases [10]–[19]. Unfortunately, increased understanding of these molecular alterations has yet to produce successful targeted therapies. We therefore performed next generation sequencing on a panel of Ewing sarcoma family tumors and cell lines to identify additional molecular alterations associated with this aggressive cancer.

## Results

To gain insight into the genetic landscape of EFT, we first performed whole genome paired-end sequencing of six Ewing sarcoma family tumors and paired constitutional DNA purified from peripheral blood. Sequencing generated an average of 375 Gb of mapped reads per sample to a mean depth of 119X, which allowed for high quality calls covering 97.7% of the genome. Additional sequencing statistics verified that the coverage and parameters used were sufficient to detect most of the sequencing variants in these samples (Table S1). To extend our findings from the whole-genome sequencing cohort, we performed targeted genomic sequencing and/or whole-transcriptome sequencing on a total of 101 EFT samples including 65 tumors and 36 cell lines, with both technologies being utilized in the majority of samples (Table S2).

### Ewing sarcoma family tumors have a low density of somatic mutation and structural variation

**Figure 1:**
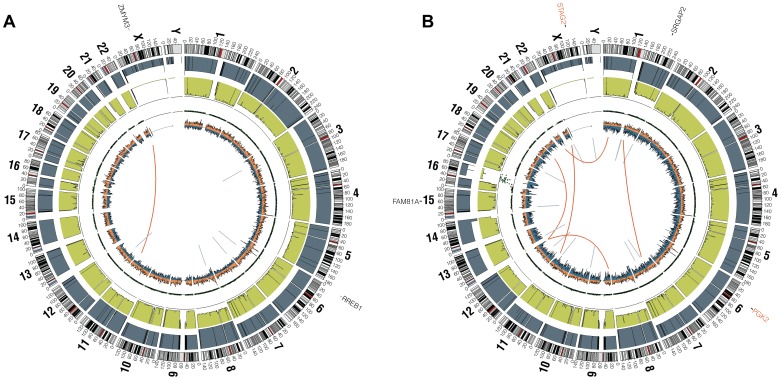
Circos plots of representative Ewing sarcoma family tumors. Circos plot tracks represent somatic mutations, from outside circle: mutated genes including missense (Black), indel (Red) and nonsense (Orange); genomic location; genome copy number alterations (Grey); lesser allele frequency (Green); LOH (dotted track); density of heterozygous SNPs (Orange); density of homozygous SNPs (Blue); Intrachromasomal (Grey) and interchromasomal (Red) rearrangements. Tumor EWS2006 (A) contains only 2 somatic coding mutations. Tumor EWS2017 (B) has 4 somatic coding mutations including a frameshift mutation in *STAG2*. Both tumors shown have the characteristic *EWSR1-FLI1* fusion and a modest degree of aneuploidy.

In the whole genome sequenced samples, we detected an average of 361 somatic mutations per tumor in non-repetitive regions and an average of 6 somatic mutations per tumor in protein coding regions (0.15 mutations/Mb of coding sequence), placing EFT at the low end of the mutation rate spectrum compared to previously reported malignancies [20]. There were no recurrent somatic small variants at the gene level within the 6 samples (Table S3). Somatic structural variants in this cohort were assessed using analysis of paired-end clones with discordant ends plus sequence coverage data from the whole genome sequencing data. Structural variants that involved a copy number change were verified using high-density SNP arrays with high degree of concordance (33/35 = 94.3%). Significant findings include previously reported alterations such as the characteristic *EWSR1-FLI1* gene fusion detected in all 6 samples, *CDKN2A* homozygous deletion in 2 samples, and frequent chromosomal gains and losses. Novel findings include multiple focal areas of loss of heterozygosity, several out-of-frame gene fusions, and a tandem-duplication within the *STAG2* gene in one sample (Table S4). There was an average of 17 structural variations per sample and no areas containing a high-density of structural variations (i.e. chromothripsis) were discovered. This number of structural variants is comparatively low relative to other pediatric tumors that have been evaluated by similar methods [21], [22]. In summary, WGS of 6 EFTs revealed the characteristic *EWSR1* fusion genes, low mutational burden and structural variations, but two tumors had loss of *STAG2* (frameshift variant in EWS2017 and focal tandem duplication in EWS2020), and two had deletion of *CDKN2A* (EWS2009 and EWS2020) (Figure 1, Figure S1).

### Tumors lacking a EWSR1-ETS fusion have distinct molecular characteristics from the remaining majority Ewing sarcoma family tumors

**Figure 2:**
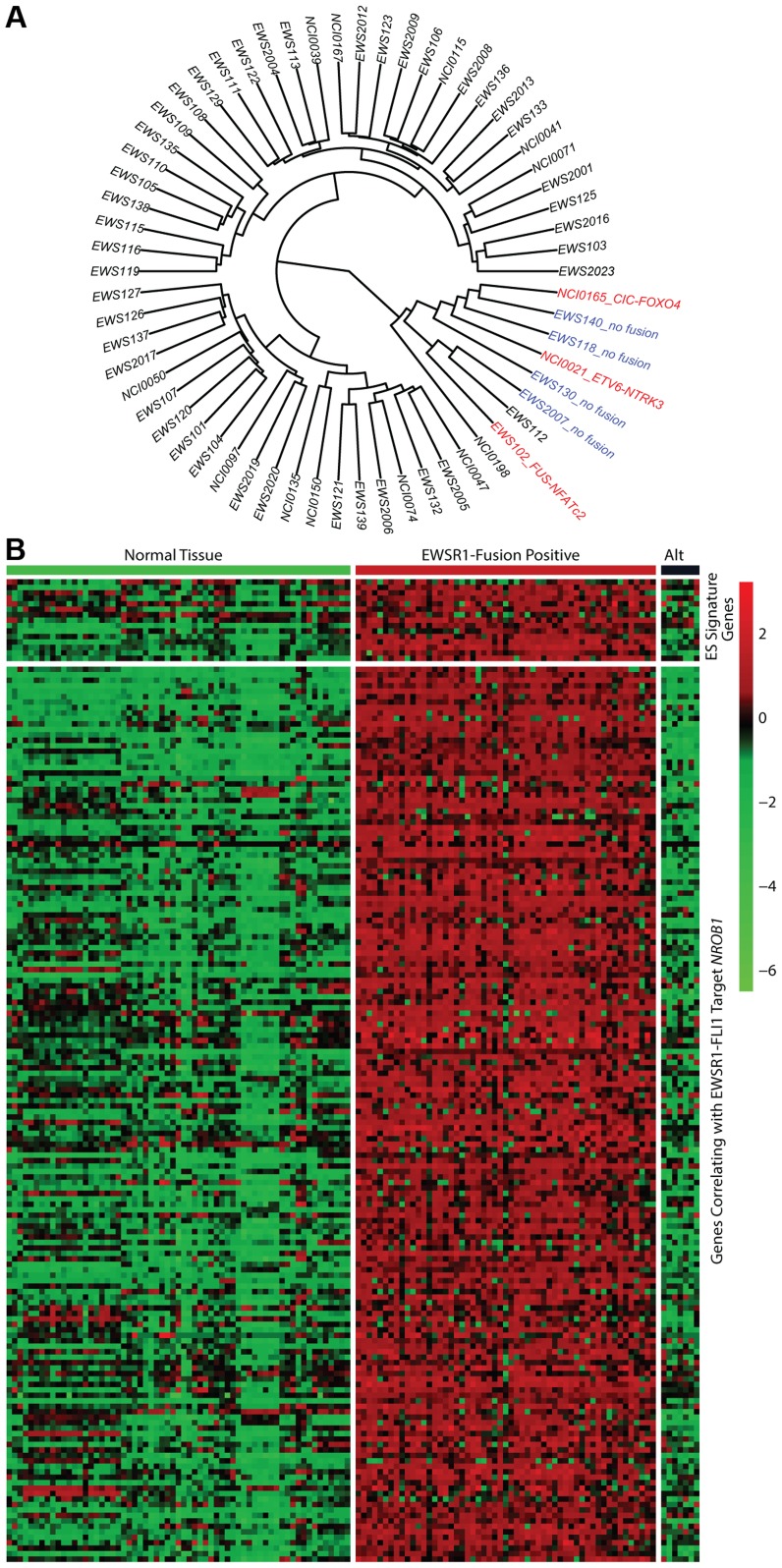
Molecular profiling of Ewing sarcoma family tumors using RNA sequencing data. EFT clinical samples that lack an EWSR1-fusion have a distinct profile. A) Hierarchical clustering based on RNA expressional profile shows the fusion negative (blue) and alternate fusion (red) samples to separate from the majority of EWSR1-fusion positive EFTs. B) Expression profile of Ewing sarcoma signature genes (top) and genes correlating with EWSR1-FLI1 target *NROB1* (bottom) in normal tissues and EFT cohort demonstrating the lack of typical expressional profile in EWSR1-fusion negative samples (Alt).

In all 31 cell lines in which RNA sequencing was performed, an EWSR1-ETS family fusion transcript was detected (Table S5). In 62 tumor samples analyzed by RNA sequencing, 55 contained an EWSR1-ETS family fusion including 28 *EWSR1-FLI1* type I (51%), 11 *EWSR1-FLI1* type II (20%), 11 other *EWSR1-FLI1* variants (20%), 3 *EWSR1-ERG* (5%), and 2 *EWSR1-FEV* fusions (4%) (Table S5). Of the 7 tumors remaining without a EWSR1-ETS fusion, one sample was found to have a novel *FUS-NCATc2* fusion (Figure S2A). Another EWSR1-ETS negative sample contained a novel *CIC-FOXO4* fusion (Figure S2B). Both of these novel fusions are in-frame and contain the functional domains of the associated genes important for oncogenic potential. A third sample contained an *ETV6-NTRK3* fusion (Figure S2C), an alteration previously reported in association with infantile fibrosarcoma, congenital mesoblastic nephromas, secretory carcinoma of breast, mammary analogue secretory carcinoma of salivary gland, and radiation-associated thyroid cancer [23]–[27]. Hierarchical clustering based on RNA expression show that the 7 tumor samples without a TET-ETS fusion, including the 3 with the above alternate fusions, cluster separately from the vast majority of EWS-ETS fusion-positive samples (Figure 2A). Additionally, these 7 samples show low expression of a collection of EWSR1-FLI1 target genes as well as low expression of a Ewing sarcoma gene signature previously reported by our group [28] (Figure 2B, Figure S3). We therefore consider these samples to be molecularly distinct from EFT and omitted them for the purposes of mutational frequency calculation. The patients with alternate fusions were noted to be clinically aggressive and to have slightly atypical histologic features, also suggesting a difference from classic Ewing sarcoma (Text S1).

### Recurrent mutation in tumor suppressor genes *STAG2*, *CDKN2A*, and *TP53*

**Figure 3:**
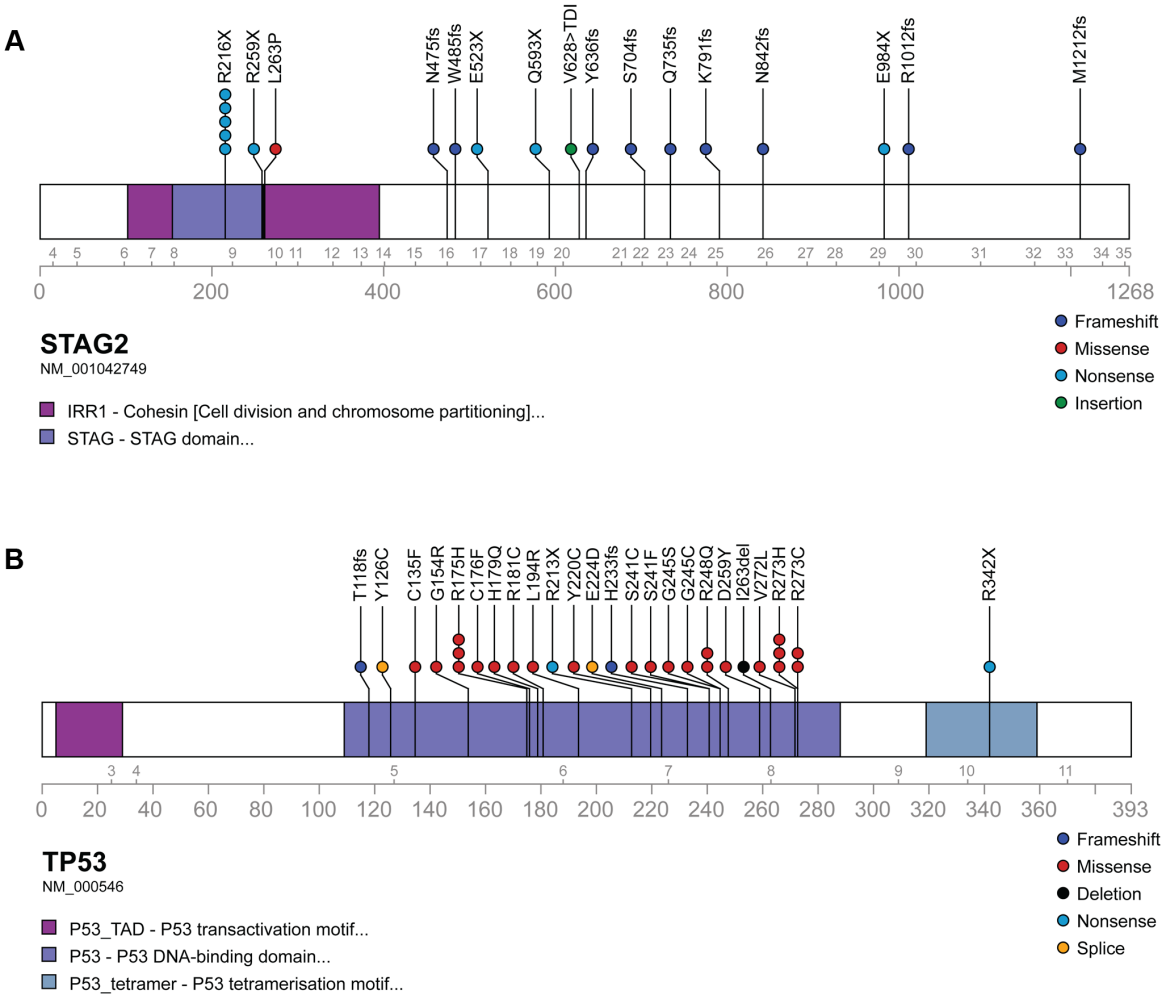
Mutational spectrum in *STAG2* (A) and *TP53* (B) on linear protein models. Exonic point mutations and small indels are shown in relation to the functional domains of these genes. Larger structural mutations and non-exonic mutations in *STAG2* are not pictured and include multi-exon intragenic deletions (3), intronic splice site mutations (3), intragenic duplication events (2), 5′ (1) and 3′ UTR (1) mutations.

**Figure 4:**
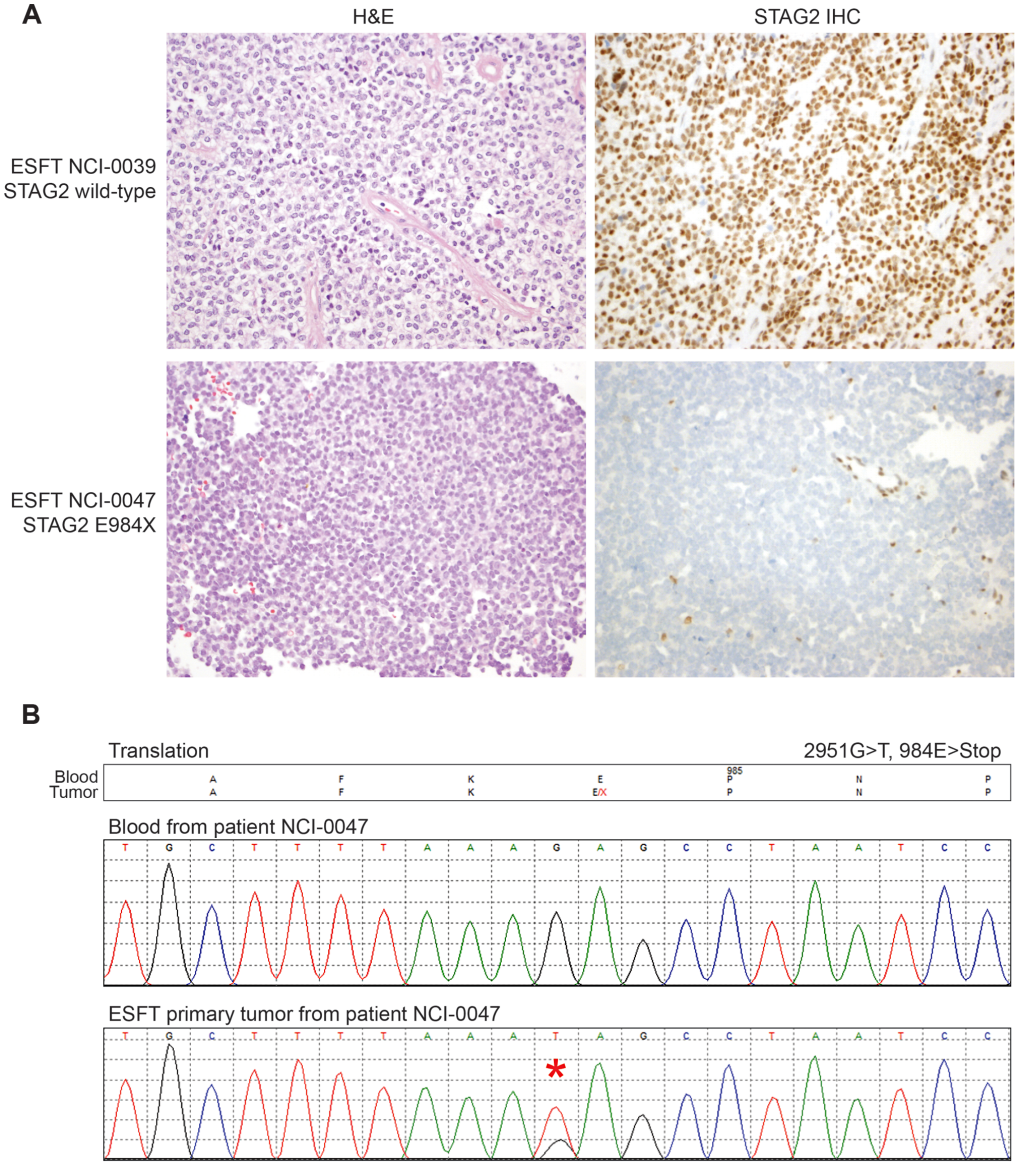
Examples of immunohistochemistry showing STAG2 expression in Ewing sarcoma tumor samples. A) STAG2 is robustly expressed in EFT harboring wild-type STAG2 alleles (top), but is completely lost in the subset of EFT harboring truncating mutations of the *STAG2* gene (bottom). Expression is retained within the non-neoplastic stromal and endothelial cells, demonstrating the somatic nature of STAG2 loss in these tumors. B) Sequence trace demonstrating the E984X *STAG2* nonsense mutation present in EFT sample NCI-0047 that is shown in A.

**Figure 5:**
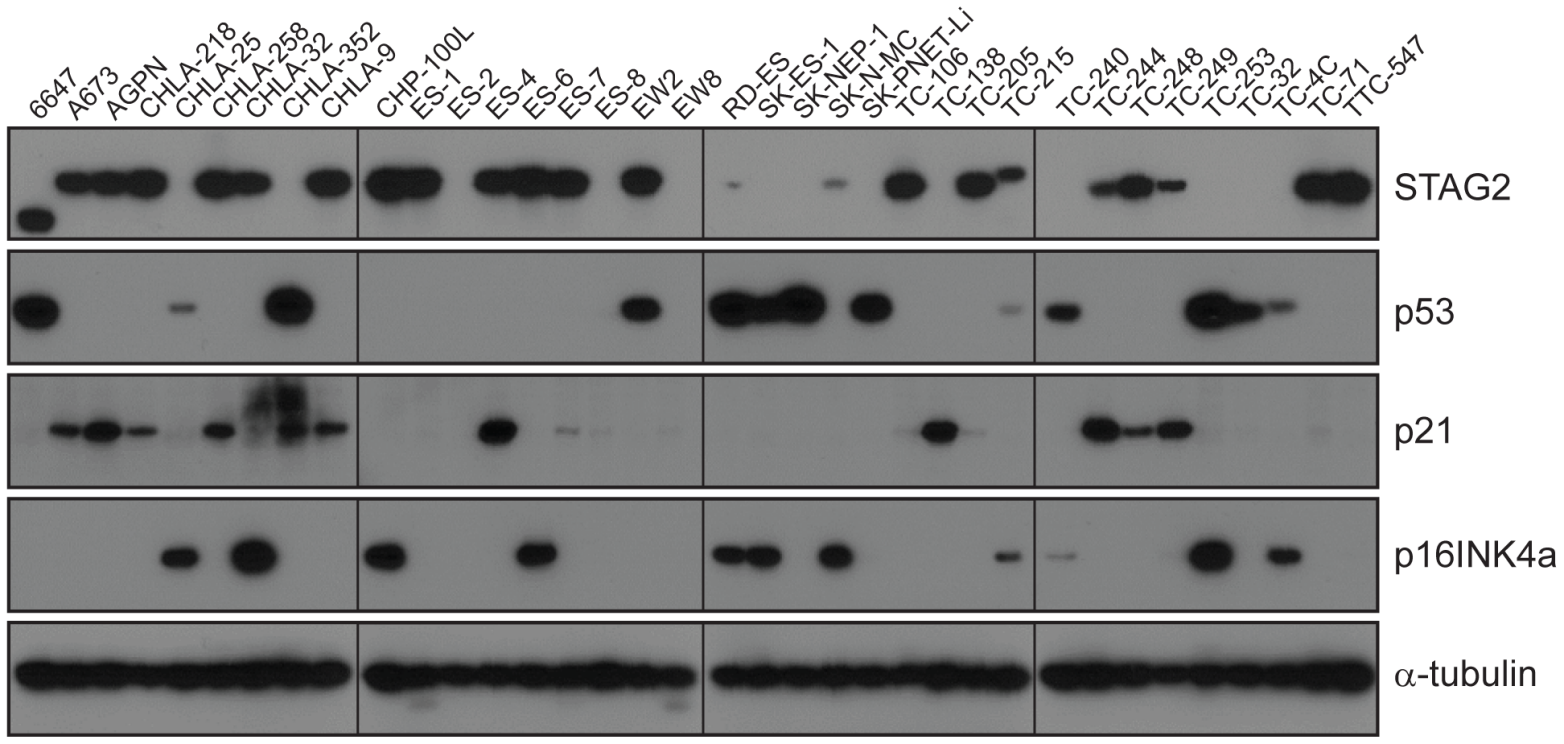
Western blots analysis of STAG2, TP53, p21^WAF1/CIP1^, and p16^INK4a^ on a panel of 36 unique EFT cell lines. 13 of 36 cell lines have complete absence of STAG2 protein, an additional two cell lines (6647 and TC-215) have STAG2 isoforms with altered molecular weight due to large intragenic in-frame insertions or deletions, one additional cell line (ES-7) has intact STAG2 expression despite a frameshift mutation occurring at amino acid residue 1212 that is C-terminal to the epitope recognized by the antibody, and two additional cell lines have intact STAG2 expression but harbor a small in-frame insertion (CHLA-9) and a missense mutation (ES-6). Absence of p16INK4a protein is seen in 25/36 cell lines including all 16 with identified *CDKN2A* deletion.

Through WGS and targeted sequencing we identified recurrent inactivating mutations of *STAG2*. To extend our genomic analysis we performed capillary sequencing of the 33 coding exons of *STAG2* in our tumor panel and in an expanded cell line panel to confirm our sequencing findings and to evaluate for additional mutations. In total, we discovered *STAG2* alterations in 30 of 101 (29.7%) of EFT samples including 14 of 65 (21.5%) clinical tumor samples and 16 of 36 (44.4%) cell lines (Table 1, Table S5). Four of these cell lines had previously been reported to harbor *STAG2* mutations [29]. Mutations were confirmed to be somatic in all tumor samples in which germline DNA from the same patient was available for comparison (7 tumors). The vast majority of the *STAG2* variants are loss of function mutations, including 10 nonsense, 8 frameshift, 3 splice-site, and 5 structural variants, as well as a 5′ deletion previously found to cause absent protein expression (Figure 3A, Table S5) [29]. The remaining three mutations of unclear functional consequence include a tumor with point mutation in the 3′ untranslated (UTR) region, a cell line with a missense mutation, and a cell line with a complex in-frame insertion (1 bp deletion replaced by a 7 bp insertion). Interestingly, 5 samples (4 tumors and 1 cell line) contained the same nonsense mutation, R216X. Mutations of *STAG2* (located on the X chromosome) were always heterozygous in samples with female genotype. In all *STAG2* mutated cell lines in which RNA sequencing data was available, the altered allele was exclusively expressed (15 cell lines), indicating in the case of female samples that the X chromosome harboring the wild type *STAG2* allele was silenced. In tumor samples, all evaluable *STAG2* mutated samples showed preferential RNA expression of the mutant allele with varying amounts of wild type allele (median variant allele frequency 0.78), likely due to varying amounts of normal tissue contamination. STAG2 mRNA expression was significantly lower in samples with truncating mutations, likely due to nonsense-mediated decay (Figure S4). Seven additional EFT samples (five tumors and two cell lines) in which *STAG2* mutation was not identified by our methods also had very low STAG2 expression comparable to samples with a truncating mutation in *STAG2* (Figure S4). Immunohistochemistry (IHC) analysis with an antibody that binds to an epitope at the C-terminus of the STAG2 protein confirmed that EFT tumors with truncating STAG2 mutations have absent STAG2 protein expression, while the admixed non-neoplastic stromal and endothelial cells had robust expression (Figure 4). Tumors with wild-type STAG2 had robust expression of STAG2 protein specifically in cell nuclei as expected (Figure 4). Tissue microarrays (TMA) from an independent cohort of genetically confirmed Ewing sarcoma cases [30] were evaluated for STAG2 expression by IHC. In 210 evaluable cases, loss of STAG2 expression was found in 30 tumors (14.3%), 28 of which demonstrated complete absence of STAG2 protein and 2 of which demonstrated mosaic loss of STAG2 (Figure S5). Western blots performed on the EFT cell line panel demonstrated complete absence of STAG2 protein in 13 cell lines and altered protein in 3 cell lines, concordant with all 14 samples with inactivating mutations and the 2 samples with low RNA expression but no identified mutation (Figure 5, Table S6).

**Table 1:** Mutational frequency of recurrently altered genes in Ewing sarcoma family tumors and cell lines.

	*STAG2*	*TP53*	*CDKN2A*
Tumors (65)	14 (21.5%)	4 (6.2%)	9 (13.8%)
Cell Lines (36*)	16 (44.4%)	23 (71.9%)	16 (50%)

*4 cell lines were tested only for *STAG2* mutation but not *TP53* nor *CDKN2A*.

We identified *TP53* mutation in 4 of 65 (6.2%) EFT tumor samples and in 23 of 32 (71.9%) EFT cell lines tested. Almost all of the *TP53* mutations we discovered are previously reported pathologic variants and/or are truncating mutations (nonsense, splice site, or frameshift) (Figure 3B, Table S5). RNA expression analysis showed that there were 4 additional EFT samples (2 tumors, 2 cell lines) in which *TP53* mutation was not identified but had low TP53 expression similar to those with a truncating mutation (Figure S6).

*CDKN2A* deletion was detected in 9 of 65 (13.8%) EFT tumors and in 16 of 32 (50%) EFT cell lines tested based on DNA and/or RNA sequencing coverage (Figure S7, Figure S8A). The semi-quantitative nature of the PCR reactions combined with varying amounts of normal contamination potentially results in a decreased sensitivity to detect this finding in our tumor samples. Western blots performed on the EFT cell lines demonstrated complete absence of p16^INK4A^ expression in all cell lines in which deletion was detected (Figure 5, Table S6).

**Figure 6:**
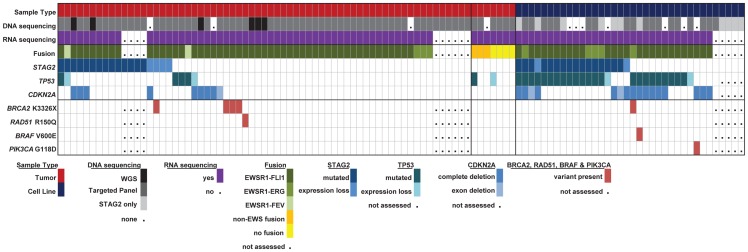
Summary of sequencing findings in EFT tumors (red) and cell lines (dark blue) highlighting recurrent alterations. There are frequent alterations in *STAG2*, *TP53*, and *CDKN2A* in EFT tumors and cell lines. 57/97 (58.7%) of samples containing an EWSR1-ETS fusion have a secondary mutation in one of these three tumor suppressor genes. Notable variants in *BRAF*, *PI3KCA*, *RAD51* and *BRCA2* are also shown.

In summary, we found that *STAG2*, *TP53* or *CDKN2A* was altered in 57 of 97 (58.7%) of EFT samples (excluding tumors lacking an EWSR1-ETS fusion) in which these 3 genes were sequenced by at least one technology (Figure 6). This count includes 26 of 65 (40.0%) clinical tumor samples and 31 of 32 (96.9%) cell lines. In the clinical tumor samples, these alterations were typically mutually exclusive in 19 of 26 (65.5%) although several samples had *STAG2* mutations in association with *TP53* mutations or *CDKN2A* deletions (Figure 6).

### High prevalence of the *BRCA2* K3326X polymorphism

In addition to the targeted gene panel that was sequenced, we examined variants in all genes from our RNA sequencing data to look for other potentially oncogenic mutations (Table S7). We discovered several well-established cancer mutations in single samples, including a *BRAF* V600E mutation (cell line A673), a *PI3KCA* mutation (cell line ES4) that has been recurrently found in multiple cancer types, and a *RAD51* alteration (tumor EWS101) associated with familial breast cancer [31]. In addition we discovered the *BRCA2* K3326X polymorphism in 5 samples, 4 from patient tumors and one in a cell line (TC-106). The patient frequency of 4 of 55 (7.3%) EFT tumor samples tested with this finding is statistically higher than expected given the population frequency of 12 of 1094 controls having this polymorphism in the 1000 genomes database (OR 7.1, p = 0.006). In our tumor samples, this polymorphism was mutually exclusive with *STAG2*, *CDKN2A*, and *TP53* mutations, though overlapped with *STAG2* expression loss in one case (Figure 6, Table S5). Germline material was not available to assess whether these findings represent germline or somatic changes in our patients. We discovered one additional *BRCA2* missense mutation (S2186T) that is previously unreported in dbSNP and is of uncertain clinical significance (Table S7). We did not identify additional altered genes in our sequencing that were predicted driver mutations.

### *STAG2* mutation is associated with alterations in the TP53 signaling pathway

A significant relationship was noted between *STAG2* loss and *TP53* mutational status in the EFT cell lines. In the 16 cell lines with deleterious *STAG2* alterations (inactivating mutation or loss of expression), there were 14 (87.5%) with *TP53* mutations, all missense. In the 20 remaining cell lines with intact *STAG2*, only 9 of the 16 (56.3%) evaluated by sequencing contained a *TP53* mutation, 5 of which were truncating and 4 of which were missense. Western blots demonstrated that 11 of 16 cell lines with deleterious *STAG2* alteration had overexpression of p53 protein (Figure 5, Table S6). Conversely, only 2 of 20 *STAG2*-intact cell lines had detectable p53. Congruent with these findings, *TP53* transcript levels from RNA sequencing were approximately 4-fold higher in cell lines with deleterious *STAG2* alteration (log2 FPKM 5.26 vs. 3.47, p = 0.0023) (Figure S9A). To assess the functional consequence of *TP53* overexpression related to *STAG2* mutation, we assessed the RNA expression levels of *CDKN1A* (which encodes p21^WAF1/CIP1^) as a marker of p53 activity. As expected, cell lines with *TP53* mutation or expression loss showed lower levels of *CDKN1A* transcript than *TP53* wild type cell lines (log2 FPKM 0.09 vs 4.85, p = 0.0005). *STAG2* mutation in the EFT cell lines similarly predicted for decreased *CDKN1A* transcript expression compared to samples without a detected mutation (log2 FPKM −0.20 vs 2.31, p = 0.018) (Figure S9B). Despite a much lower rate of concordant *TP53* mutation, there was also a significant association between *STAG2* mutation and decreased *CDKN1A* transcript expression in the tumor cohort (log2 FPKM 4.08 vs 5.18, p = 0.039) (Figure S9C) and a trend towards increased TP53 expression (log2 FPKM 5.51 vs. 5.38, p = 0.19). In summary, *STAG2* mutation was associated with higher *TP53* and lower *CDKN1A* expression in both tumors and cell lines and associated with more frequent missense *TP53* mutations in cell lines.

### STAG2 loss and clinical outcome in EFT patients

Clinical characteristics of the tissue microarray cohort were evaluated in relation to STAG2 IHC status. The 210 evaluable cases included 154 primary tumors, 46 recurrent/metastatic samples, and 10 tumors with limited clinical information (Table S8). STAG2 expression loss was more common in recurrent/metastatic samples than primary samples, though this difference did not reach statistical significance (21.7% vs. 12.3%, p = 0.15). In 110 primary tumor samples in which clinical outcomes data was available, there was a trend towards a modest decrease in overall survival in patients whose tumors had STAG2 loss (p = 0.10); this evaluation was limited, however, by small numbers of patients with STAG2 negative tumors in the analysis (Figure S10). Clinical information from the sequencing cohort was also analyzed, though survival information was not available. We found no significant differences in age, gender, stage, or primary tumor site (extremity vs. non-extremity) between *STAG2* mutated and wild-type samples, though numbers were small in these comparisons (Table S9).

## Discussion

To our knowledge, this is the first and largest report to utilize next-generation sequencing technology to characterize the genomic landscape of Ewing sarcoma family of tumors and evaluate for recurrent mutations. We find a very low somatic mutational rate in EFT compared to most previously reported tumor types. We hypothesize this to be the case for multiple reasons. First, it appears that a number of pediatric tumor subtypes tend to have lower mutation rates than those reported in adult cancer [21], [32]–[34]. This may be due in part to the shorter amount of time that the precursor cell has to accumulate passenger mutations during normal cell division but may also represent a fundamental difference common to pediatric cancers. For example, it is possible that pediatric cancers may be more epigenetically driven compared to adult cancers and therefore require a lesser genetic-level contribution to oncogenesis. Second, the low mutation rate of Ewing sarcoma even amongst several reported pediatric cancer types may reflect a fundamental characteristic of fusion-driven cancers. This is in keeping with differences noted between fusion-positive and fusion-negative rhabdomyosarcomas reported by our group and others [22], [35].

Interestingly, we found by RNA sequencing that a significant number (11%) of our tumor samples that were pathologically diagnosed as Ewing sarcoma family tumors lacked a characteristic TET-ETS fusion and appeared to be molecularly distinct from EFT by expression profile. Within this group, we report two novel fusions, *FUS-NCATc2* and *CIC-FOXO4*, that are in-frame and may be oncogenic based on available literature regarding the function of the genes involved. *NCATc2* is a non-ETS family transcription factor that has recently been described as an alternate fusion partner to *EWSR1* in a small series of “Ewing sarcoma-like” tumors [36], but has not previously been reported to partner with the alternate TET family member *FUS*. *CIC* gene rearrangements, particularly *CIC-DUX4* fusions, have been described in a group of aggressive undifferentiated small blue round cell sarcomas thought to be distinct from Ewing sarcoma [37]. *FOXO4*, a forkhead family transcription factor, has been described as an uncommon fusion partner to *MLL* in acute leukemias [38] and as a rare *PAX*-gene fusion partner in rhabdomyosarcoma [39]. We discovered one additional fusion, *ETV6-NTRK3*, which has been reported in other cancer types but not in EFT [23]–[27]. Whether these tumors should be considered as a variant of EFT or a distinct entity is debatable. Our RNA data suggests that these alternate fusion samples, as well as the other TET-ETS fusion negative samples, have a distinct expression pattern from the other EFT tumors and do not match well to a previously reported EFT expression signature [28]. Practically, the rarity of these variants amongst an already uncommon disease will make clinical distinction difficult.

In our survey for genetic alterations, we discovered *STAG2* mutations in 21.5% of Ewing sarcoma family tumor samples and 44.4% of EFT cell lines tested, the vast majority of which are inactivating mutations. STAG2 protein detection by IHC in an independent tumor cohort showed STAG2 loss in 14.3% of tumors. While immunohistochemistry will identify all tumors with homozygous deletions and truncating mutations, it will not detect tumors harboring missense mutations, in-frame insertions or deletions, or duplication events. This may help to explain the small discrepancy between our sequencing and immunohistochemical analyses. *STAG2* mutation has previously been reported in one Ewing sarcoma tumor and in multiple EFT cell lines [29] and has additionally been reported as a recurrently mutated tumor suppressor gene in other tumor types including glioblastoma, urothelial carcinoma, and acute myeloid leukemia [29], [40]–[44].

Mutations in *TP53* and *CDKN2A* were found in frequencies similar to that previously reported [10]–[19]. In total we found that 40% of EFT clinical tumors and 97% of EFT cell lines have disruption of *STAG2*, *TP53* or *CDKN2A*. The striking difference in mutational frequencies between tumors and cell lines, particularly in *TP53*, may be a result of culture conditions and the process of immortalization. Despite these frequency differences, the increased molecular characterization of a large selection of EFT cell lines evaluated in this study provides an invaluable resource for further study.

In addition to the recurrent mutations in *STAG2*, *TP53* and *CDKN2A*, we found a high prevalence of the *BRCA2* K3326X polymorphism, seen in 7.3% of our clinical tumor samples. Occurring in approximately 1% of the general population, this premature stop codon has not been shown to confer an increased risk of breast or ovarian cancer [45] and is classified as a benign variant by the International Agency for Research on Cancer Unclassified Genetic Variants Working Group [46]. In contrast, groups studying lung [47], pancreatic [48], and squamous esophageal cancers [49] have all reported a significantly increased rate of this polymorphism in the germline DNA of patients with these cancer types. In our cohort, as only tumor material was evaluated for this finding, we could not distinguish whether this was a germline or somatic change. Further study is warranted to clarify this aspect and to confirm the association.

*STAG2* encodes a subunit of cohesin, a structural protein complex involved in chromosomal organization and so named due to its function of creating “cohesion” between sister chromatids after DNA replication. In addition to *STAG2*, other recurrent alterations in subunits of this complex have been reported across a number of cancer types [42], [50], [51]. Potentially, the oncologic mechanism for cohesin mutation is disrupted chromosomal segregation during mitosis leading to accumulation of structural mutations and aneuploidy [29]. Though we find EFT to have a low rate of aneuploidy overall in our comprehensively characterized WGS cohort, further work is indicated to clarify whether or not a *STAG2* mutation is linked to increased aneuploidy in this tumor histology. Cohesin is also known to play a regulatory role in transcription [52] and is essential for recombinant-based DNA repair mechanisms [53], though it remains to be seen if and how much each of these essential cellular processes are responsible for the oncologic transformation resulting from cohesin deficit. In our evaluation of the cellular impact of *STAG2* in EFT, we note a significant intersection of *STAG2* mutation with alteration of the *TP53* pathway. We find a strong correlation between *STAG2* loss and overexpression of p53 in EFT cell lines. We note that this overexpressed p53 protein very frequently contains a pathogenic missense mutation. *STAG2* mutated samples also had low RNA expressional levels of *CDKN1A* (encoding p21^WAF1/CIP1^), a well-established mediator of p53 tumor suppressor activity [54]. Taken together these data suggest that transcriptional dysregulation of the p53-p21 axis may play a role in *STAG2*-mediated oncogenesis, at least in EFT cell lines. Though there was less overlap between *STAG2* mutation and *TP53* mutation in the sequenced tumor cohort, we noted the same pattern of decreased *CDKN1A* expression in *STAG2* mutant samples.

We found STAG2 loss to be more common in cell lines than tumors, more frequent in metastatic or recurrent disease than primary tumors, and to be associated with a trend towards modestly decreased survival. Given the significant percentage of tumors harboring a *STAG2* mutation in this cancer type, further investigation into the oncogenic mechanism, clinical consequence, as well as strategies for directed therapy are warranted. For example, preclinical data suggest that cohesin deficiency may increase sensitivity to poly(ADP–ribose) polymerase (PARP) inhibition [55], a drug class that has also been identified by systematic screening to be effective in Ewing sarcoma cell lines [56], and that is undergoing clinical testing in this tumor type. Additionally, future sequencing efforts should be extended to evaluate for alternate routes to cohesin deficiency in EFT.

This study demonstrates that at least a subset of Ewing's sarcoma is not a single hit disease driven solely by a EWS-ETS fusion gene, but rather is a genetically complex disease which harbors additional recurrent genetic alterations that likely contribute to the pathogenesis of EFT. Further studies will be needed to determine if the presence of these additional genetic aberrations will impact the sensitivity/resistance to small molecule inhibitors of EWS-FLI1 or PARP that are currently in development and early phase clinical trials.

## Materials and Methods

### Tissue processing

All specimens for sequencing were obtained from patients with appropriate consent from the local institutional review board in accordance with the Children's Oncology Group and the National Cancer Institute. Clinical samples were obtained from collaborations with the Cooperative Human Tissue Network, the Children's Hospital of Westmead, Australia, the Children's Oncology Group, and the National Institutes of Health Clinical Center. Tumors were classified as a Ewing sarcoma family tumor by a sarcoma pathologist and the host institution using standing histological techniques. Fifty-two tumors were from the primary disease site and had not been exposed to previous treatment. Fifteen tumors were from recurrent/metastatic sites and for five tumors we lacked this clinical information. Clinical and pathological data for the sequencing cohort are summarized in Table S9. Tumor samples were evaluated by a pathologist for the presence of more than 70% tumor content before DNA/RNA extraction and sequencing. DNA was extracted from qualifying tumor samples and matched blood using either AllPrep Mini (Qiagen) or Agencourt Genefind v2 (Beckman Coulter) DNA extraction kits. RNA was extracted using the RNeasy Micro Kits according to the manufacturer's protocol (Quiagen). Genotyping confirmed independence of these samples.

### EFT cell lines

All EFT cell lines used in the study underwent short tandem repeat (STR) profiling for independence testing and all were confirmed to have a unique profile. This characterization is described in detail in Table S10.

### EFT tissue microarray

Tissue microarrays were obtained from an independent cohort of genetically confirmed Ewing sarcoma cases [30]. The associated clinical information is summarized in Table S8.

### Whole genome sequencing

Whole genome paired-end sequencing was performed using the Complete Genomics platform. Data analysis was accomplished using the CGA tools package v2.0 [57], ANNOVAR v2012-05-25 [58], and Circos v0.52 [59] in build hg19. Somatic variants were determined first by comparison to the matched normal DNA. To remove artifacts specific to the sequencing platform, we eliminated any somatic variants also found in normal samples [50 in-house samples and 69 Complete Genomics samples (http://www.completegenomics.com/public-data/69-Genomes/)]. The Somatic Score (http://media.completegenomics.com/documents/DataFileFormats+Cancer+Pipeline+2.0.pdf) is based on a Bayesian model and takes account of read depth, base call quality, mapping/alignment probabilities, and measured priors on sequencing error rate for both the germline and tumor variants. Verification by Sanger sequencing was performed on all high-confidence somatic variant calls (by default Somatic Score> = −10) affecting protein coding or a splice site (SNVs, substitutions, insertions, deletions), including 55 SNVs. We determined that more stringent somatic score cut-off was required in our cohort to achieve adequate positive predictive value of variants calls, likely due to the low mutation rate in our tumor type. Relative to all high-confidence variant calls we established a sensitivity of 86.7% and specificity of 90.7% for a Somatic Score cut-off of 3. Somatic mutations at or above this score and all verified mutations with lower scores were used for further analysis.

The Complete Genomics somatic copy number segmentation is based on 2-kb windows and utilizes coverage in the matched germline sample for normalization of the tumor sample coverage. Lesser allele fraction (LAF) calculations are based on allele read counts in the tumor at loci that are called heterozygous in the matched germline sample. In addition to default filtering done by the Complete Genomics segmentation algorithm, copy number variants were considered high-confidence if they were either large (> = 10 kb AND containing > = 10 heterozygous SNPs for LAF calculation) OR highly altered (homozygous deletions and focal amplifications > = 5 copies) OR supported by somatic junction(s) (ex: junction detected spanning both ends of a region of LOH). Somatic junctions were called using CGAtools and junctions were filtered by footprints smaller than 70 bases, less than 10 discordant mate pairs, under-represented repeats, and presence in the baseline set of 69 Complete Genomics genomes. We additionally filtered junctions that were present in 50 in-house germline DNA samples that were sequenced on the same platform.

### Targeted genomic sequencing

Genomic sequencing was performed using a custom multiplex PCR designed to include the entire coding sequence of the majority of altered genes in the whole-genome sequencing discovery cohort as well as *TP53* and in total encompassing 106.3 kB of target region (Table S11). Primers for the targeted sequencing were designed using the Ion Ampliseq designer (Life Technologies) and sequencing was performed on the IonTorrent PGM (Life Technologies). PGM sequencing data was analyzed using Torrent Suite software v3.2 (Life Technologies) and ANNOVAR. Genomic sequencing was performed to an average mean coverage of 311× and variants were called using high-confidence thresholds and filtered to include only those that are protein altering and unreported or rare (population allele frequency <0.005) in the dbSNP and 1000 genomes databases. Mutations of interest were verified by capillary sequencing with a <5% false positive rate. Sequence coverage data was calculated at a position, exon and gene levels to look for structural alterations of the recurrently mutated genes (Figure S8). Coverage data was visualized using the Integrated Genomics Viewer.

### RNA sequencing

PolyA selected RNA libraries were prepared for sequencing on the Illumina HiSeq2000 using TruSeq v3 chemistry according to the manufacturer's protocol (Illumina). RNA sequencing was performed with an average yield of 18.6 Gb per sample. Raw reads were mapped using to ENSEMBL reference (hg19) using TopHat2.0 [60]. Fusion analysis was done using TopHat 2.0 and DeFuse 0.6 [61]. The 3 alternated fusions described were confirmed using RT-PCR using flanking primers and Sanger sequencing of the resultant product.

Expression FPKM results were obtained at both gene and transcript level using CuffLinks 2.1 [62]. The log2 FPKM expression results from TopHat mapping were median-normalized using in-house data from 63 normal tissue samples. Exon level expression was calculated using the formula RPKM = (r * 10^9^)/(f * R), with r being the number of reads mapped to an exon, f being the exon length, and R being the total read count of the sample. Hierarchical clustering was performed on normalized log2 FPKM expression values at the gene level using Euclidean distance and Ward agglomeration method.

For variant detection, samtools (http://samtools.sourceforge.net/) is used to count the number of reads uniquely mapped to a position found as variant in DNA sequencing of the same sample or a position of interest based on a mutation being present in the TCGA (http://cancergenome.nih.gov/) or compared to the reference genome hg19 in genes of interest. If there are reads supporting a variant base then the total reads supporting it are counted and variant allele frequency is calculated.

### SNP array

SNP arrays were performed on the 6 tumor whole genome sequencing cohort to confirm copy number findings. SNP array analysis was conducted on HumanOmni2.5 or HumanOmni5 arrays (Illumina) and the data were analyzed with GenomeStudio (Illumina) and Nexus Copy Number v7 (Biodiscovery Inc.). Copy number state and allelic ratio was manually assessed in all areas of copy number variation and structural variation predicted by WGS and was concordant with WGS prediction in 33/35 (94%).

### Sanger sequencing of *STAG2* gene

Individual exons of STAG2 were PCR amplified from genomic DNA using the conditions and primer pairs previously described [29]. PCR products were purified using the Exo/SAP method followed by a Sephadex spin column. Sequencing reactions were performed using BigDye v3.1 (Applied Biosystems) using an M13F primer and were analyzed on an Applied Biosystems 3730×l capillary sequencer. Sequences were analyzed using Mutation Surveyor (SoftGenetics). Traces with putative mutations were reamplified and sequenced from both tumor and matched normal DNA from blood when available.

### STAG2 immunohistochemistry

A mouse monoclonal antibody to STAG2 from Santa Cruz Biotechnology (clone J-12, sc-81852) was used at a dilution of 1∶100. Immunostaining was performed in an automated immunostainer (Leica Bond-Max) following heat-induced antigen retrieval for 30 min in high pH epitope retrieval buffer (Bond-Max). Primary antibody was applied for 30 min, and Bond-Max polymer was applied for 15 min. Diaminobenzidine was used as the chromogen, and samples were counterstained with hematoxylin. Samples in which both the tumor and normal cells failed to stain for STAG2 were considered antigenically non-viable and were excluded from the analysis.

### Western blot analysis

Primary antibodies used were STAG2 clone J-12 (Santa Cruz Biotechnology), p53 clone 7F5 (Cell Signaling), p16 (BD Pharmingen #554079), p21 clone DCS60 (Cell Signaling), and α-tubulin Ab-2 clone DM1A (Neomarkers). Protein was isolated from EFT cell lines in RIPA buffer, resolved by SDS-PAGE, and immunoblotted following standard biochemical techniques.

### Statistical testing

For the BRCA2 K3326X polymorphism, a two-tailed Fisher Exact Test was used to calculate p-value for the Odds Ratio significantly different from 1. For RNA expressional analysis, a two-tailed student T Test assuming unequal variances was used to calculate a p-value for difference in population means. For tissue microarrays, the p-value for differences in frequency of STAG2 mutation in primary and recurrent/metastatic samples was calculated using two-tailed Fischer Exact Test. P-value for association of STAG2 expression with overall survival was calculated using univariate analysis.

## Supporting Information

Figure S1 Circos plots of remaining whole genome sequencing cohort. Circos plot tracks represent somatic mutations, from outside circle: mutated genes including missense (Black), indel (Red) and nonsense (Orange); genomic location; genome copy number alterations (Grey); lesser allele frequency (Green); LOH (dotted track); density of heterozygous SNPs (Orange); density of homozygous SNPs (Blue); Intrachromasomal (Grey) and interchromasomal (Red) rearrangements. Tumors EWS2008 (A), EWS2009 (B), EWS2012 (C) and EWS2020 (D) all demonstrate low numbers of coding mutations and modest amounts of structural variation.
(PDF)

Figure S2 RNA sequencing reads highlighting novel fusions. A) *FUS-NFATc2* fusion in sample EWS102. RNA sequencing generated 65 high-quality reads spanning this junction. The resulting transcript is an in-frame fusion joining exon 6 of *FUS* and exon 9 of *NFATc2*. B) *CIC-FOXO4* fusion in sample NCI0165. RNA sequencing generated 355 high-quality reads spanning this junction. The resulting transcript is an in-frame fusion joining exon 20 of *CIC* and exon 2 of *FOXO4*. C) ETV6-NTRK3 fusion in sample NCI0021. RNA sequencing generated 64 high-quality reads spanning this junction. The resulting transcript is an in-frame fusion joining exon 5 of *ETV6* and exon 13 of *NTRK3*. All three junctions were verified by RT-PCR followed by Sanger sequencing.
(PDF)

Figure S3 Detailed expression profile of Ewing sarcoma signature genes (top, starred) and genes correlating with EWSR1-FLI1 target *NROB1* (bottom) in normal tissues and EFT cohort demonstrating the lack of typical expressional profile in EWSR1-fusion negative samples (right) **Indicates gene is both part of Ewing sarcoma gene signature and correlates with *NROB1*.
(PDF)

Figure S4 Exon-level RNA expression of STAG2 in Ewing sarcoma family tumors (A) and cell lines (B) shown by median normalized z-score. Samples with truncating mutation correlate with low levels of expression. Cell lines 6647 and TC-4C show distinct pattern of contiguous multi-exon expression loss consistent with deletion of these exons. Tumor samples EWS108, EWS112, EWS135 and NCI0198 and cell lines SK-NEP-1 and TC-138 have low expressional levels comparable to samples with truncating mutation despite absence of identifiable genetic alteration.
(PDF)

Figure S5 STAG2 immunohistochemistry in Ewing sarcoma tissue microarrays. STAG2 is robustly expressed in the majority of samples (left) but expression is completely lost in a subset of tumors (right). In STAG2 negative samples, expression is retained within the non-neoplastic stromal and endothelial cells, demonstrating the somatic nature of STAG2 loss in these tumors.
(PDF)

Figure S6 Exon-level RNA expression of TP53 in Ewing sarcoma family tumors (A) and cell lines (B) shown by median normalized z-score. Samples with a truncating mutation show low levels of expression. Tumor samples EWS109 and NCI0071 and cell lines ES-6 also have low expressional levels comparable to those samples with a truncating mutation despite absence of identifiable genetic alteration. Cell line SK-N-MC has loss of expression of contiguous exons, suggesting deletion affecting that region.
(PDF)

Figure S7 Exon-level RNA expression of CDKN2A in Ewing sarcoma family tumors (A) and cell lines (B) shown by median normalized z-score. Homozygous deletion of *CDKN2A* is demonstrated by near-zero expression of this gene across all exons. Tumor EWS125 and cell lines 6647 and CHLA-9 have focal loss of expression of a single exon.
(PDF)

Figure S8 DNA sequencing coverage from the targeted sequencing approach was used to detect copy number alterations in recurrently mutated genes. A) Coverage in *CDKN2A* relative to average sequencing coverage in the same sample shows outlier samples predicted to have homozygous deletion of the gene. In a subset of samples, copy number status was assessed by SNP array to verify that *CDKN2A* was correctly predicted as deleted (green) or wild type (red). B–D) Copy number alterations seen in STAG2 based on normalized sequencing coverage plotted against genomic position on the X chromosome. B) Cell line 6647 contains a heterozygous deletion containing the 1^st^–11^th^ coding exons of *STAG2*. C) Cell line TC-4C has a hemizygous deletion in the 5^th^–12^th^ coding exons of *STAG2*. D) Cell line TC-215 has an intragenic duplication event as evidenced by doubling of copy number across contiguous exons.
(PDF)

Figure S9 Box and whisker plots showing range and percentiles of RNA expression (log2 FPKM) in subgroups determined by *STAG2* status. A) EFT cell lines with *STAG2* loss have increased TP53 expression. B–C) EFT cell lines (B) and tumors (C) with *STAG2* mutation have decreased CDKN1A expression.
(PDF)

Figure S10 Cumulative overall survival of patients with primary, non-metastatic tumors in the TMA cohort stratified by STAG2 IHC status. This analysis demonstrates a trend towards decreased survival in patients whose tumors have loss of STAG2 expression.
(PDF)

Table S1 Sequencing statistics for whole genome sequencing cohort.
(XLSX)

Table S2 Overview of sequencing studies performed.
(XLSX)

Table S3 Confirmed somatic mutations in whole genome sequencing cohort.
(XLSX)

Table S4 High confidence structural variants in whole genome sequencing cohort.
(XLSX)

Table S5 Overview of mutational findings and fusion status in sequencing cohort.
(XLSX)

Table S6 Summary of Western blot experiments in 36 EFT cell lines with comparison to genomic findings.
(XLSX)

Table S7 Selected variants from RNA sequencing analysis, including the BRCA2 K3326X polymorphism.
(XLSX)

Table S8 STAG2 expressional status in tissue microarray cohort as determined by immunohistochemistry.
(XLSX)

Table S9 Clinical characteristics of sequencing cohort.
(XLSX)

Table S10 STR profiles of cell lines in analysis.
(XLSX)

Table S11 Primers used for targeted sequencing approach.
(XLSX)

Text S1 Clinical course and pathological description of tumors that harbor an alternate fusion.
(DOCX)
