# Correction: The Genomic Landscape of the Ewing Sarcoma Family of Tumors Reveals Recurrent STAG2 Mutation

**DOI:** 10.1371/journal.pgen.1004629

**Published:** 2014-08-11

**Authors:** The PLOS Genetics Staff

There are several errors in the published manuscript.

1. There are 4 instances where NFATc2 is misspelled as NCATc2; in the 6^th^ line of the abstract, in the first paragraph of the subsection “Tumors lacking a EWSR1-ETS fusion have distinct molecular characteristics from the remaining majority Ewing sarcoma family tumors” in the Results section and twice in the second paragraph of the Discussion section.

2. Authors Julia Gerard and Jund-Sik Kim are listed as being affiliated to the Tumour Bank, The Children’s Cancer Research Unit, The Children’s Hospital at Westmead. Both of these authors should instead be affiliated with The Department of Oncology, Lombardi Comprehensive Cancer Center, Georgetown University School of Medicine.

3. The flowing sentence should also have been included in the acknowledgements section: “The authors thank the Children’s Oncology Group Ewing Sarcoma Biology Committee for specimens obtained via Human Specimen Banking Grant U24 CA114766”.
