# Supplementary figures and images for: The Genomic Landscape of the Ewing Sarcoma Family of Tumors Reveals Recurrent STAG2 Mutation
Source: PLoS Genet. 2014 Jul 10;10(7):e1004475. doi: 10.1371/journal.pgen.1004475 (PMC4091782; doi:10.1371/journal.pgen.1004475)

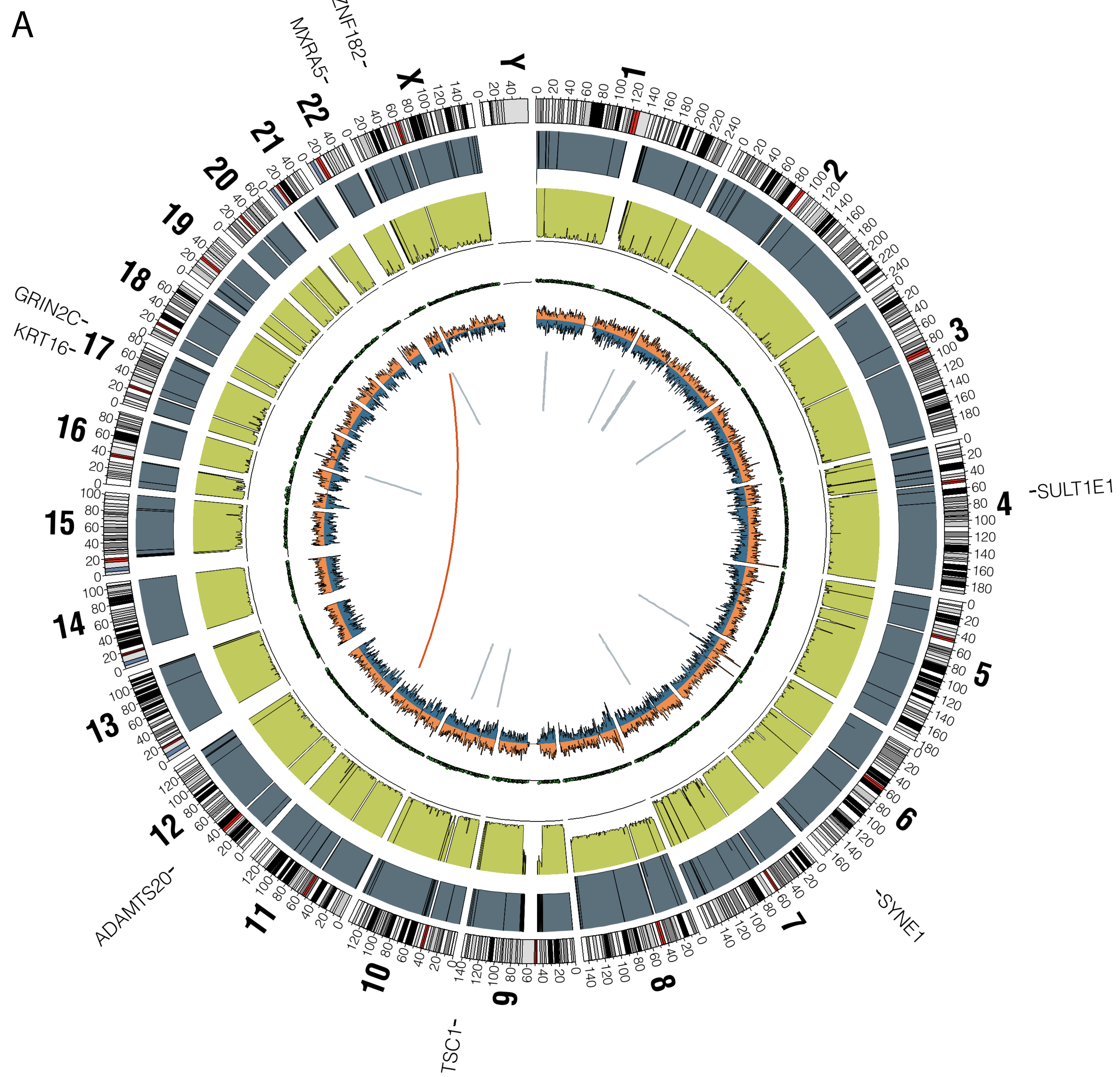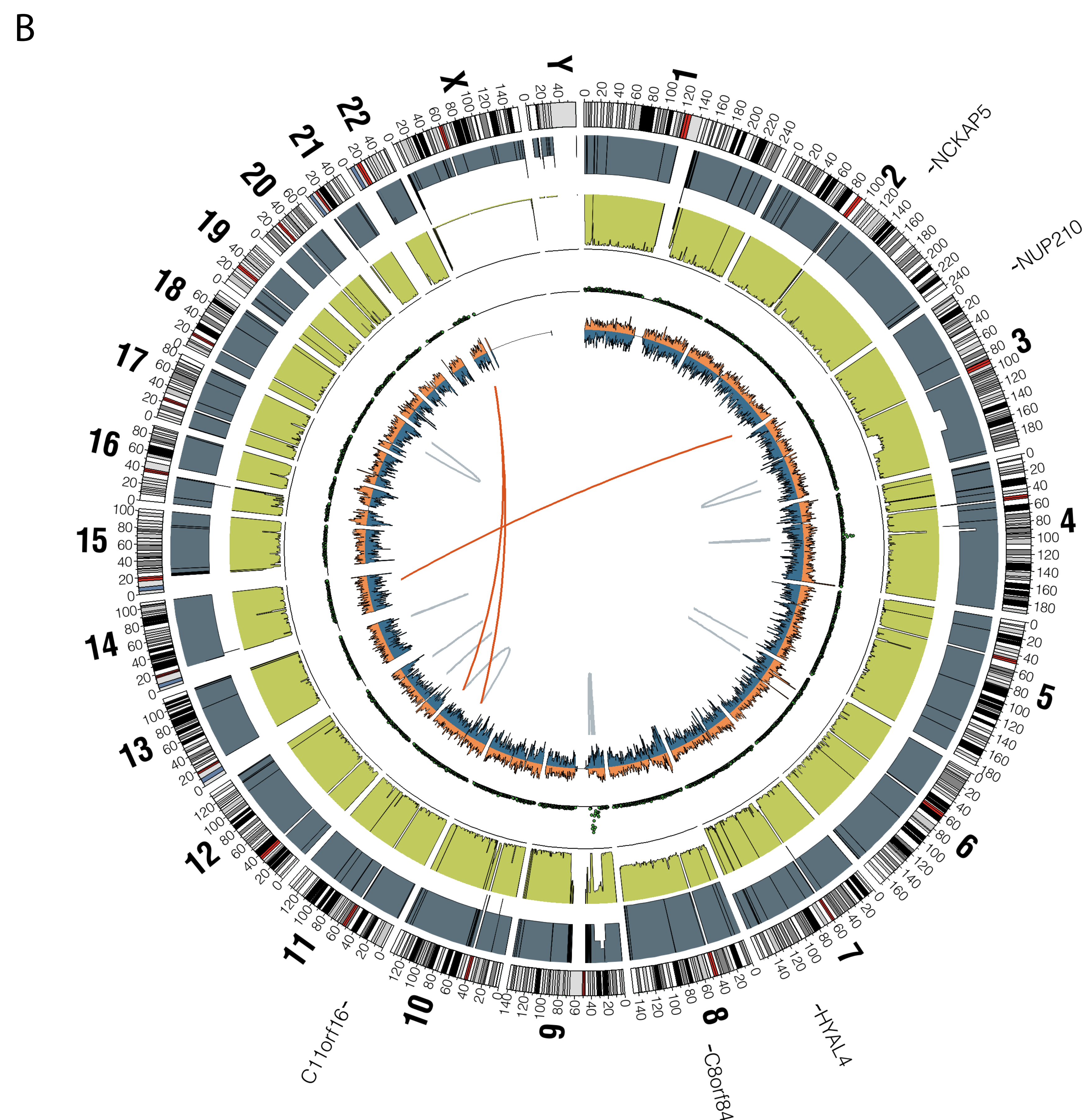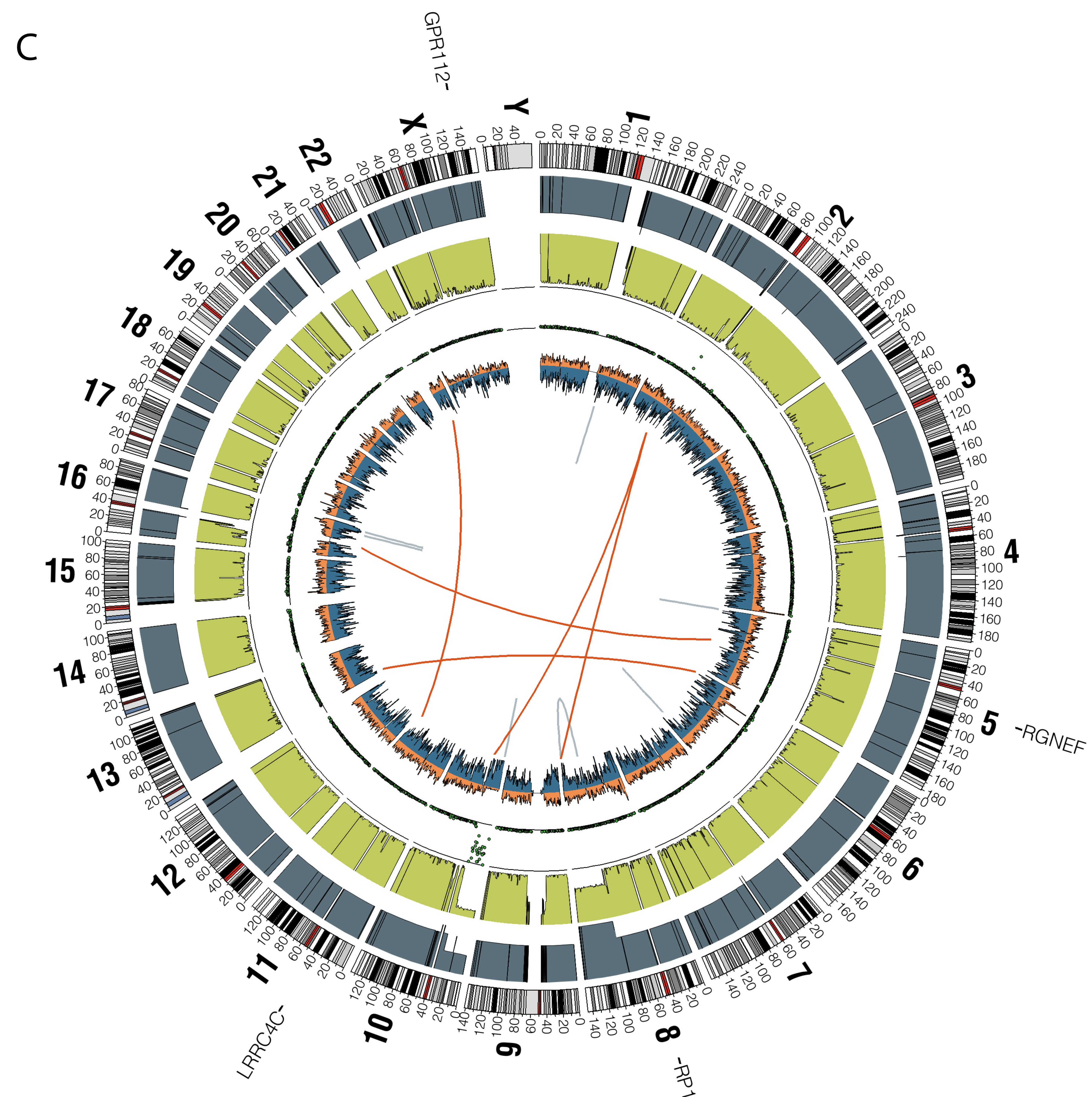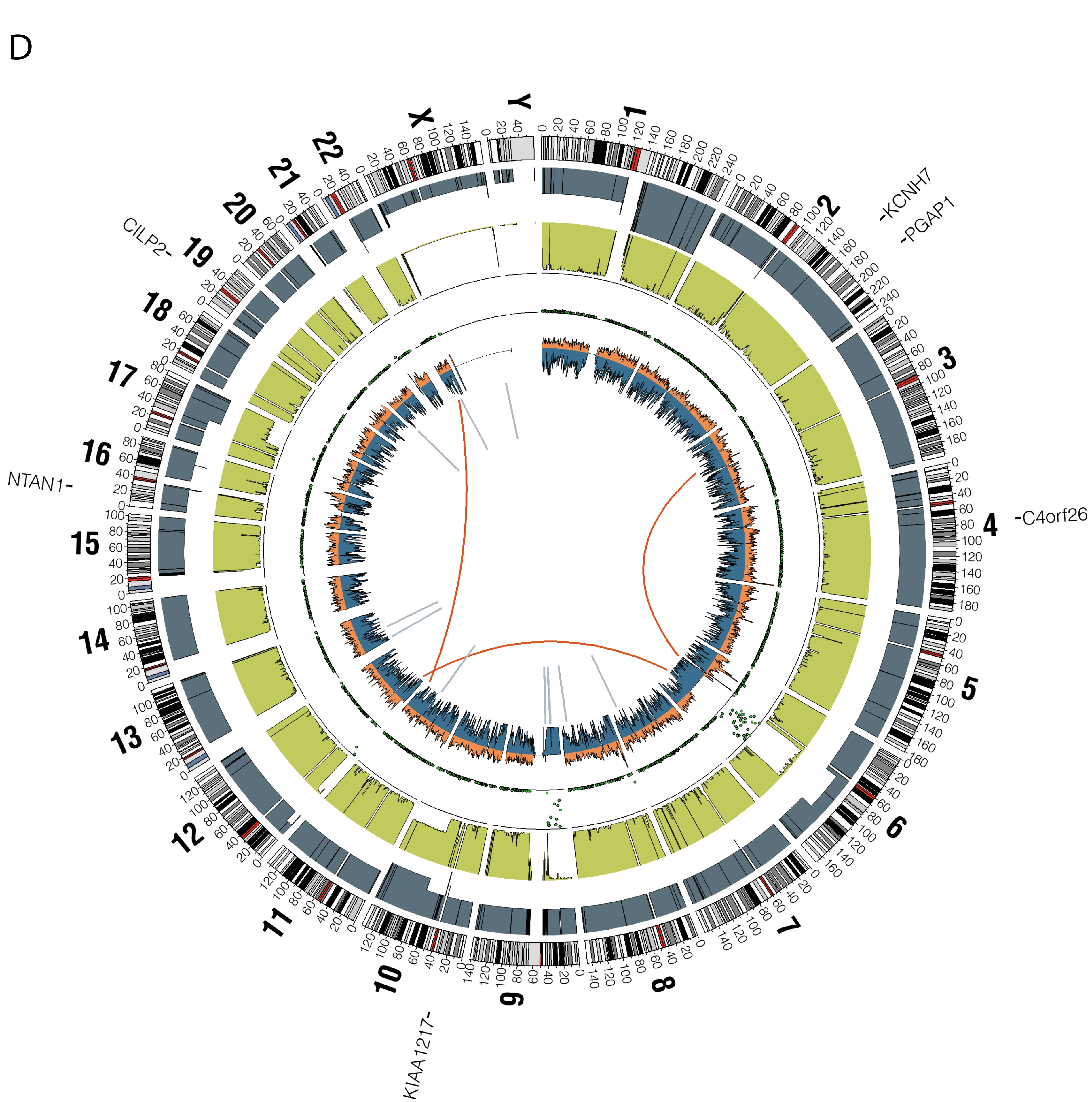

Supplement: Figure S1 — Circos plots of remaining whole genome sequencing cohort. Circos plot tracks represent somatic mutations, from outside circle: mutated genes including missense (Black), indel (Red) and nonsense (Orange); genomic location; genome copy number alterations (Grey); lesser allele frequency (Green); LOH (dotted track); density of heterozygous SNPs (Orange); density of homozygous SNPs (Blue); Intrachromasomal (Grey) and interchromasomal (Red) rearrangements. Tumors EWS2008 (A), EWS2009 (B), EWS2012 (C) and EWS2020 (D) all demonstrate low numbers of coding mutations and modest amounts of structural variation. (PDF) [file pgen.1004475.s001.pdf]

## A

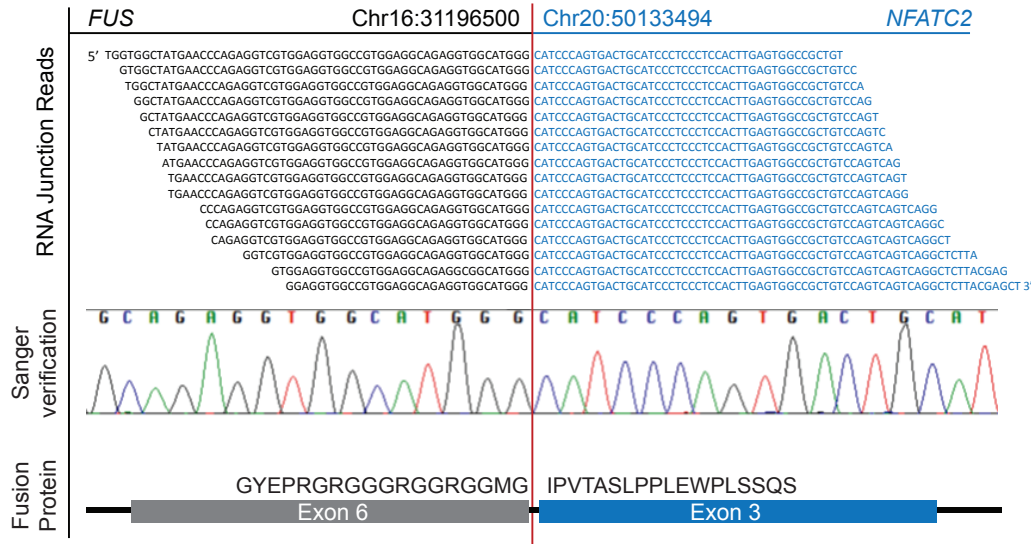

## B

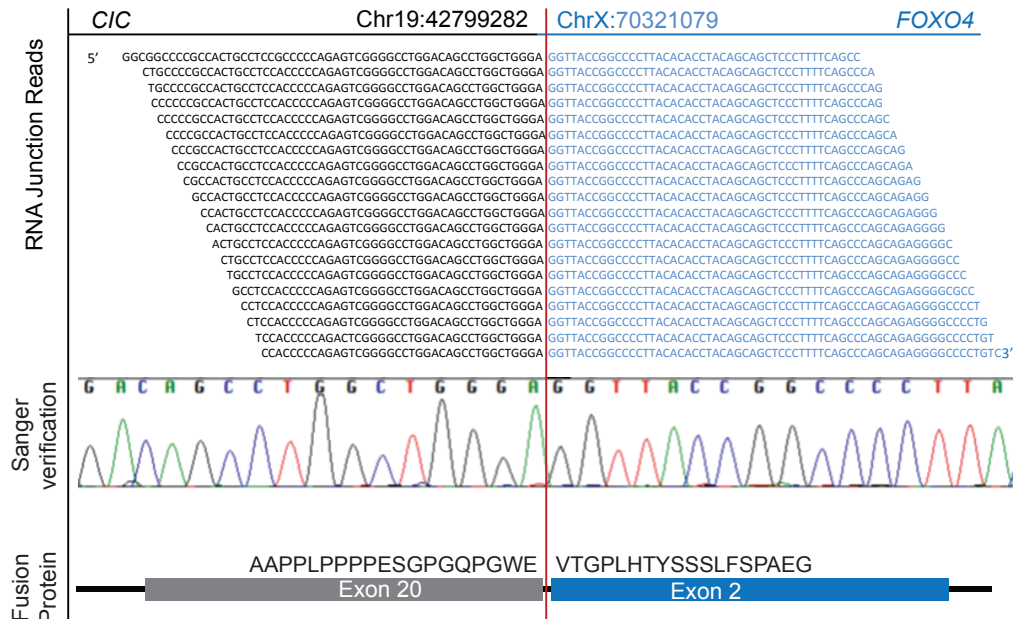

## NCI0021

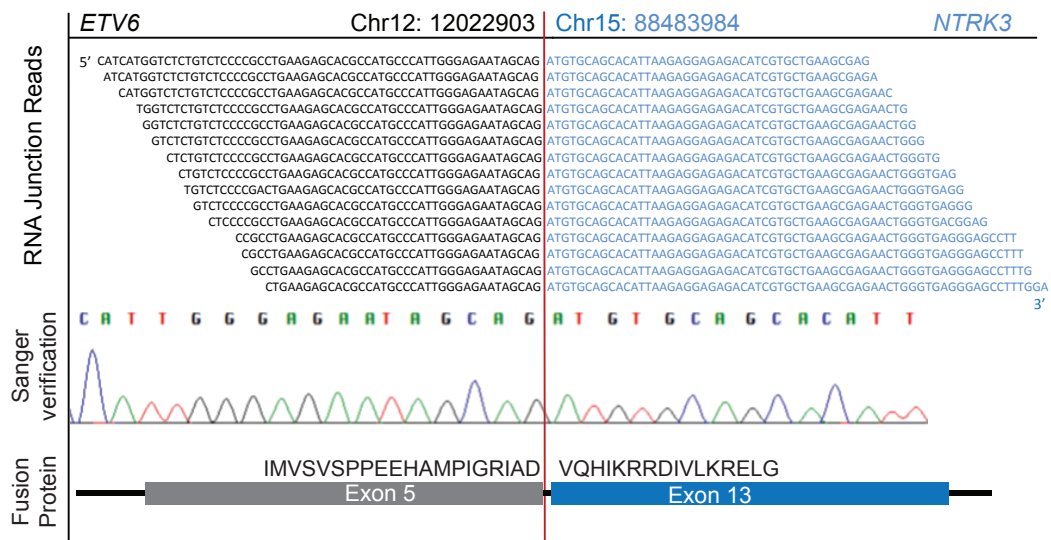

Supplement: Figure S2 — RNA sequencing reads highlighting novel fusions. A) FUS-NFATc2 fusion in sample EWS102. RNA sequencing generated 65 high-quality reads spanning this junction. The resulting transcript is an in-frame fusion joining exon 6 of FUS and exon 9 of NFATc2. B) CIC-FOXO4 fusion in sample NCI0165. RNA sequencing generated 355 high-quality reads spanning this junction. The resulting transcript is an in-frame fusion joining exon 20 of CIC and exon 2 of FOXO4. C) ETV6-NTRK3 fusion in sample NCI0021. RNA sequencing generated 64 high-quality reads spanning this junction. The resulting transcript is an in-frame fusion joining exon 5 of ETV6 and exon 13 of NTRK3. All three junctions were verified by RT-PCR followed by Sanger sequencing. (PDF) [file pgen.1004475.s002.pdf]

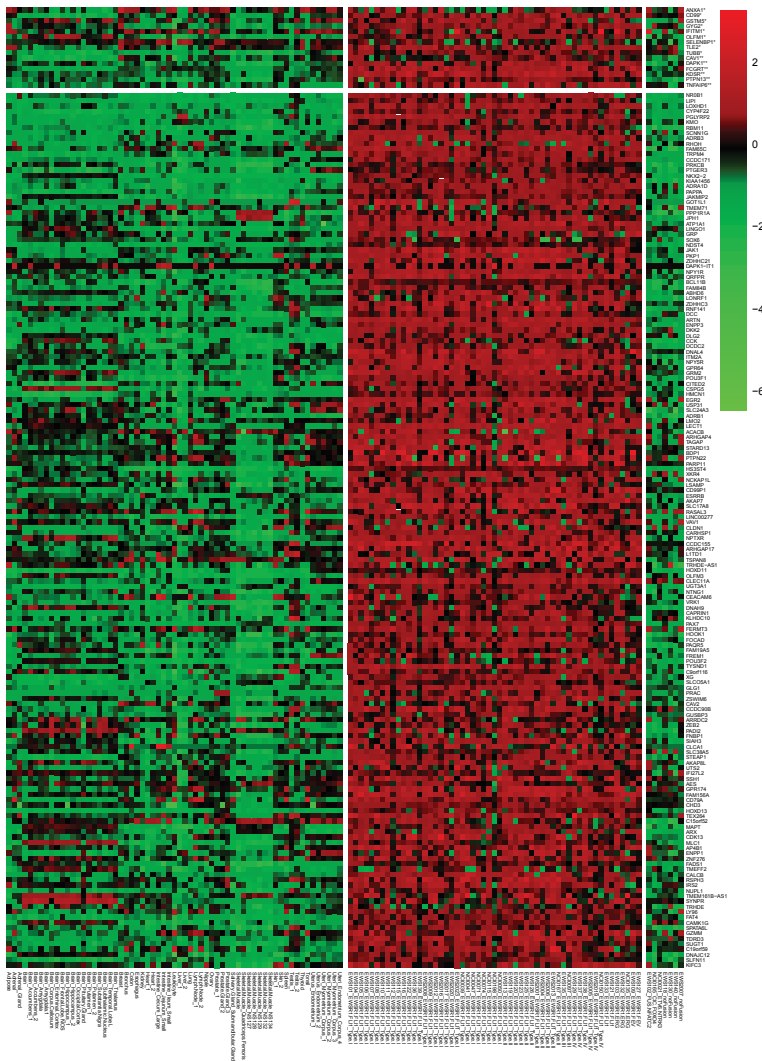

Supplement: Figure S3 — Detailed expression profile of Ewing sarcoma signature genes (top, starred) and genes correlating with EWSR1-FLI1 target NROB1 (bottom) in normal tissues and EFT cohort demonstrating the lack of typical expressional profile in EWSR1-fusion negative samples (right) **Indicates gene is both part of Ewing sarcoma gene signature and correlates with NROB1. (PDF) [file pgen.1004475.s003.pdf]

**A**

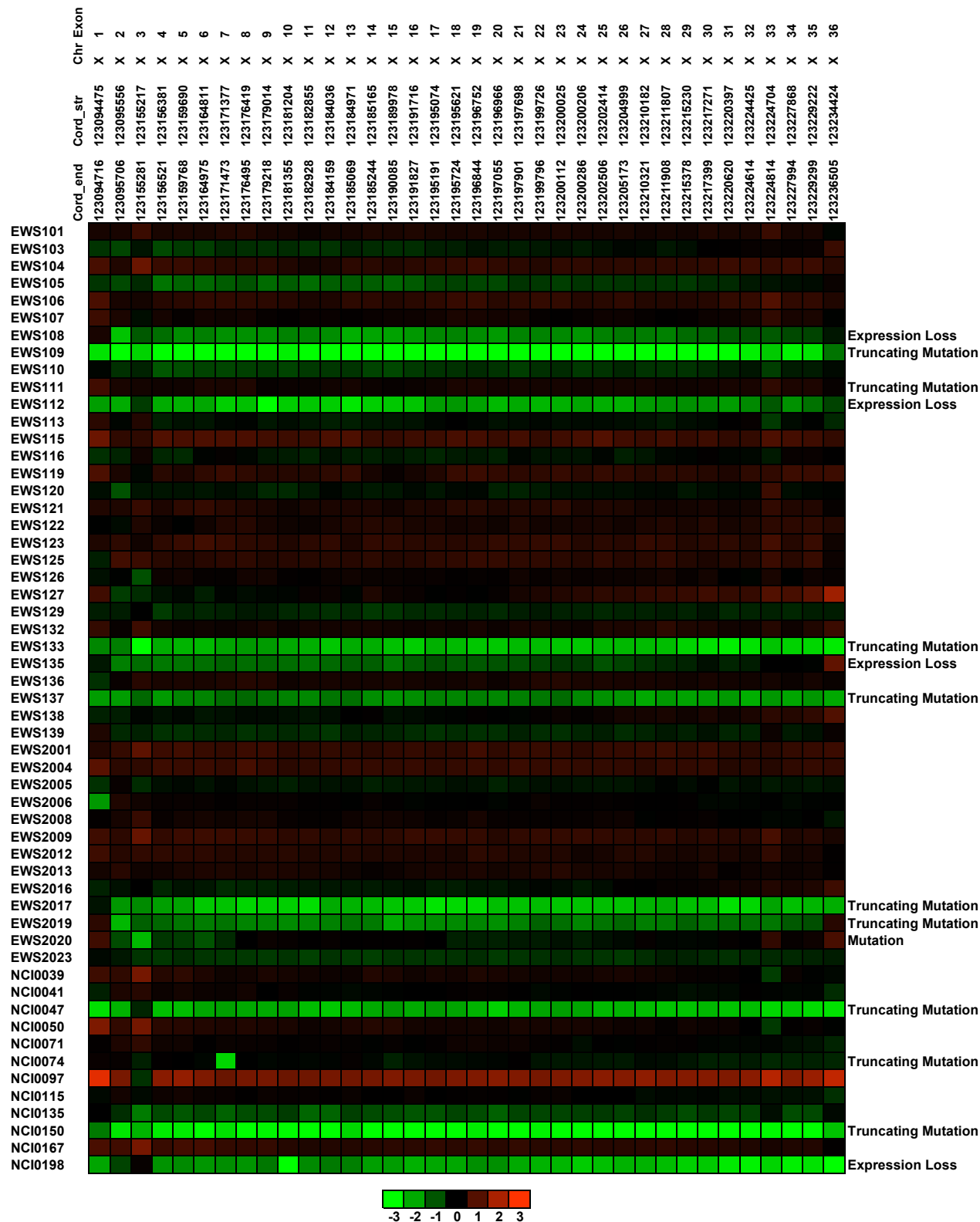

# B

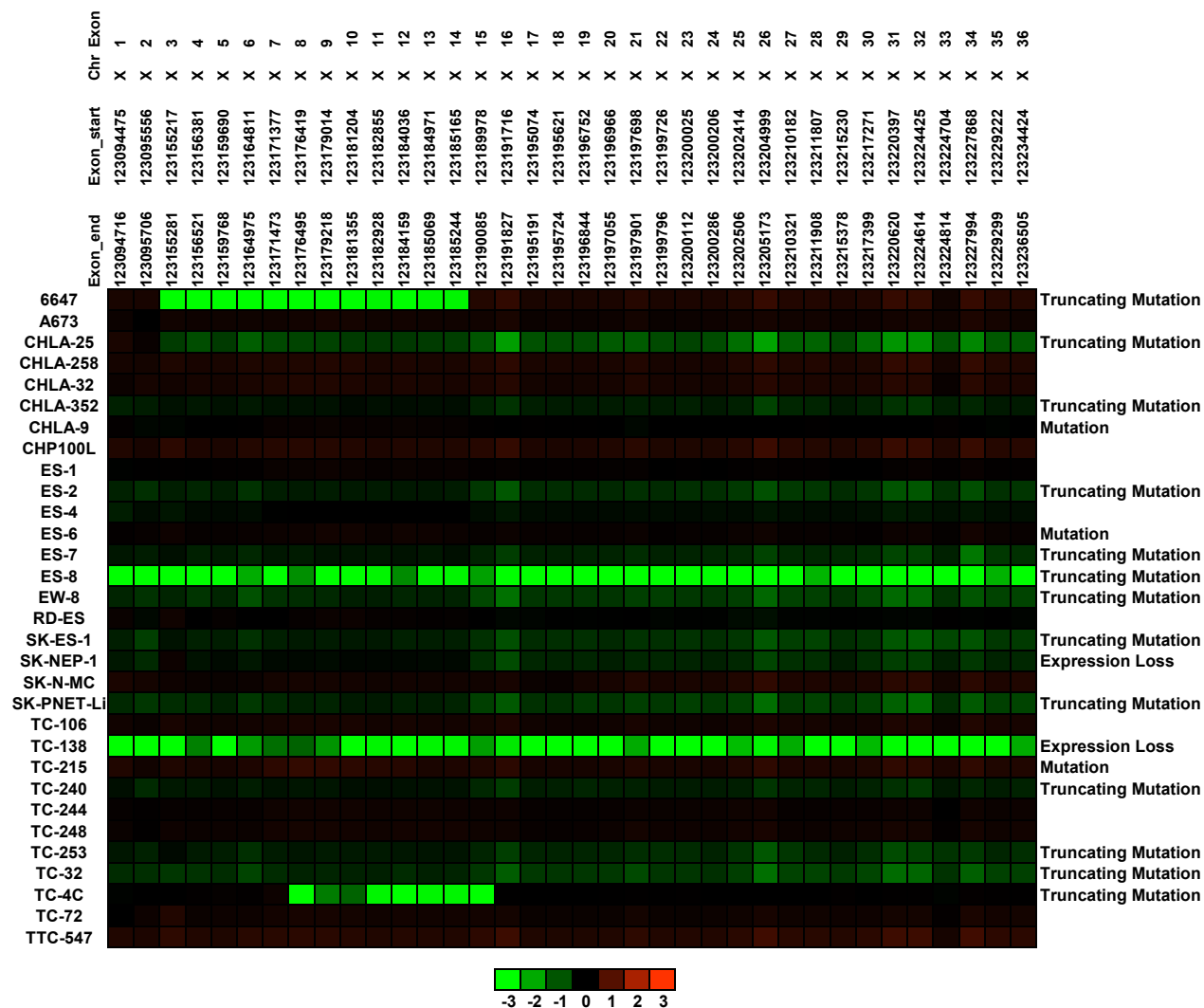

Supplement: Figure S4 — Exon-level RNA expression of STAG2 in Ewing sarcoma family tumors (A) and cell lines (B) shown by median normalized z-score. Samples with truncating mutation correlate with low levels of expression. Cell lines 6647 and TC-4C show distinct pattern of contiguous multi-exon expression loss consistent with deletion of these exons. Tumor samples EWS108, EWS112, EWS135 and NCI0198 and cell lines SK-NEP-1 and TC-138 have low expressional levels comparable to samples with truncating mutation despite absence of identifiable genetic alteration. (PDF) [file pgen.1004475.s004.pdf]

A

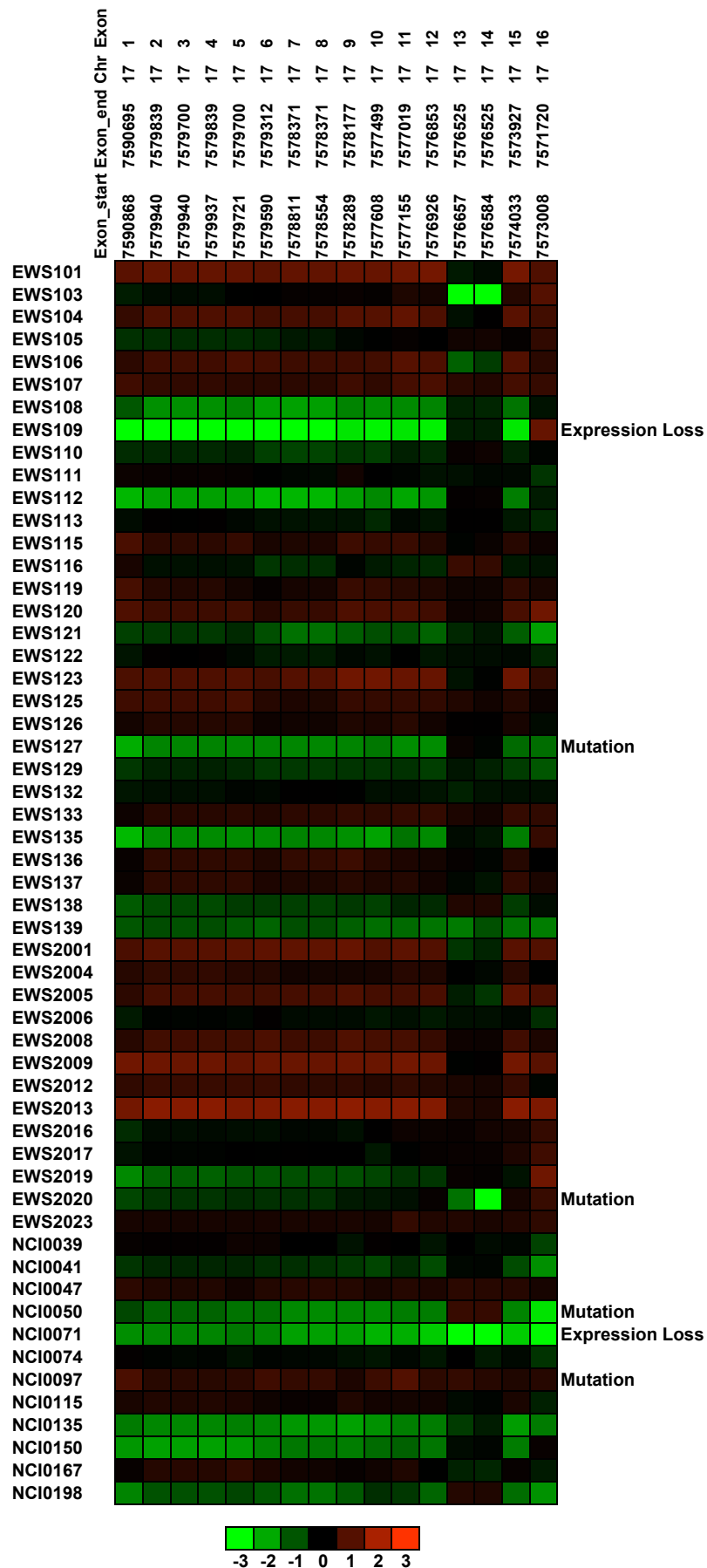

B

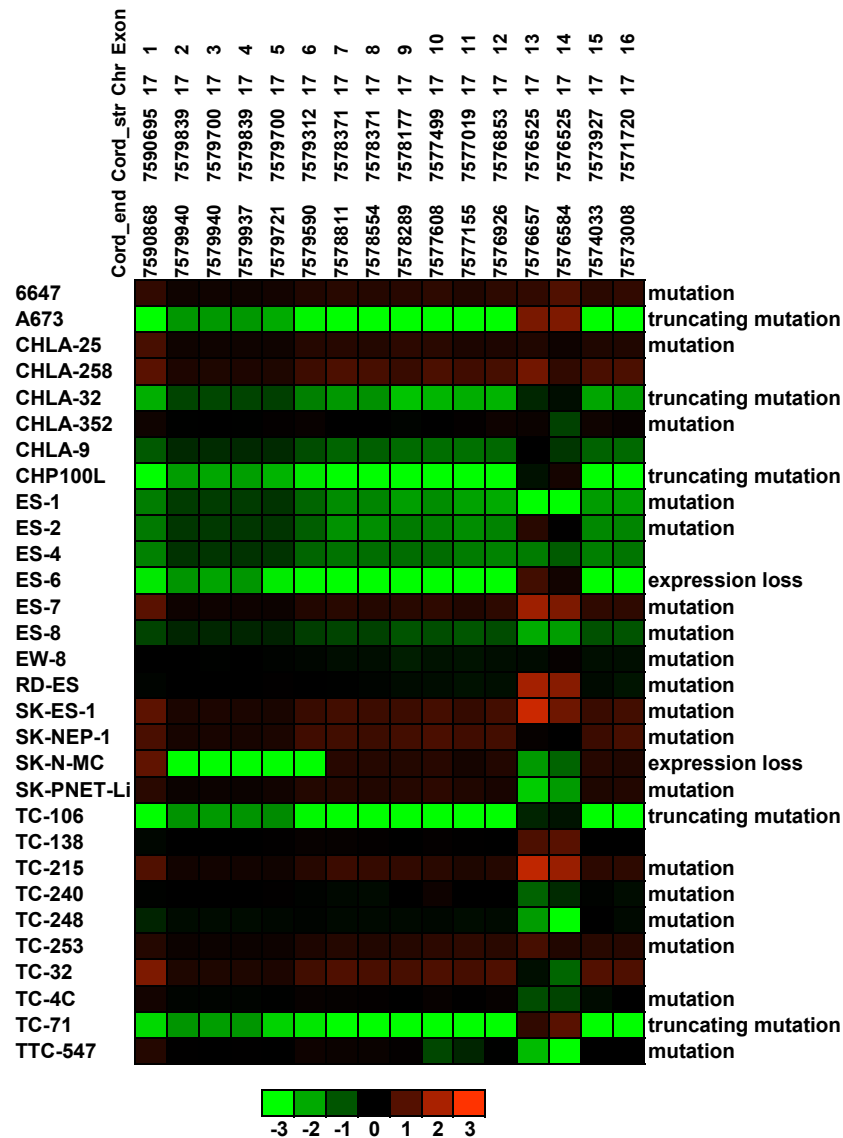

Supplement: Figure S6 — Exon-level RNA expression of TP53 in Ewing sarcoma family tumors (A) and cell lines (B) shown by median normalized z-score. Samples with a truncating mutation show low levels of expression. Tumor samples EWS109 and NCI0071 and cell lines ES-6 also have low expressional levels comparable to those samples with a truncating mutation despite absence of identifiable genetic alteration. Cell line SK-N-MC has loss of expression of contiguous exons, suggesting deletion affecting that region. (PDF) [file pgen.1004475.s006.pdf]

A

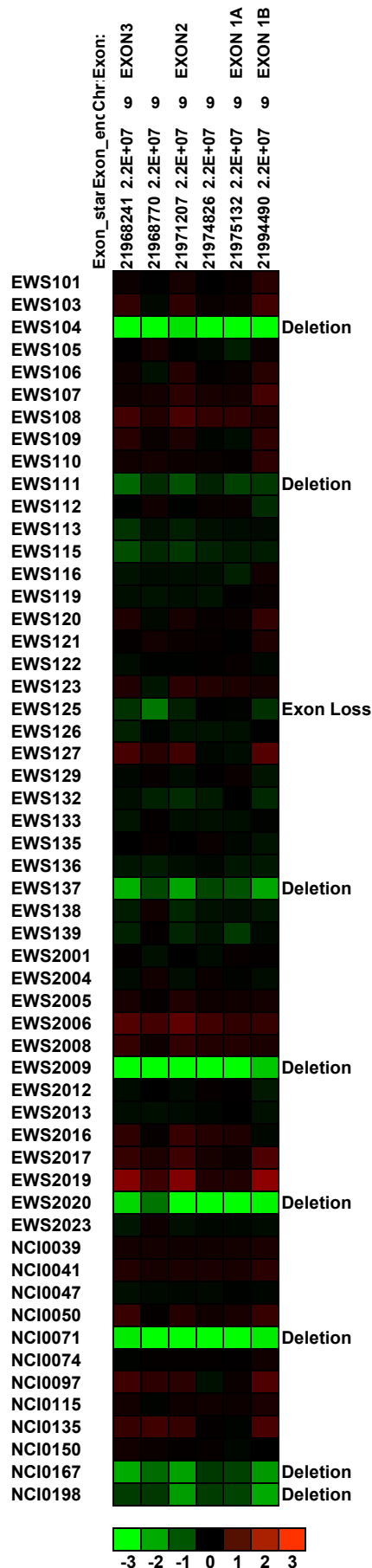

B

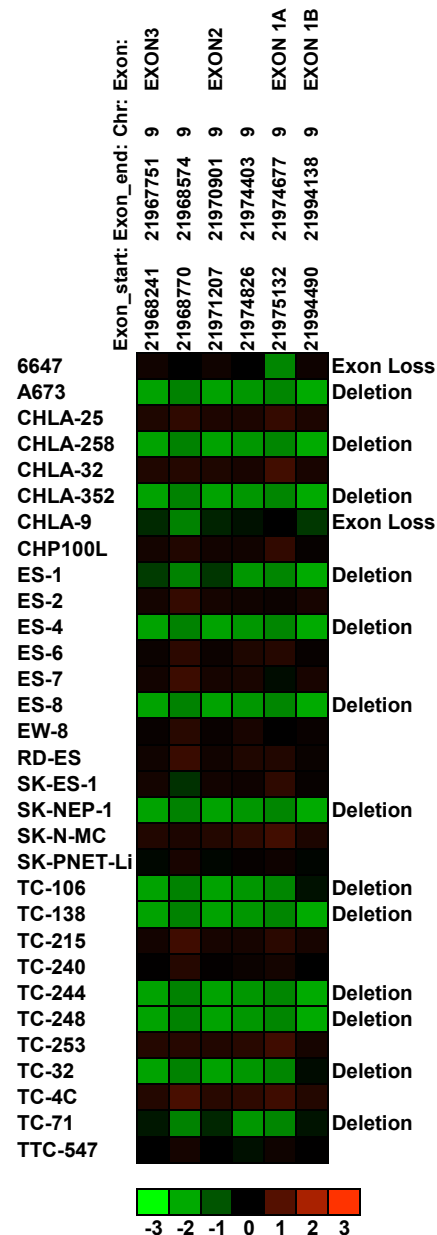

Supplement: Figure S7 — Exon-level RNA expression of CDKN2A in Ewing sarcoma family tumors (A) and cell lines (B) shown by median normalized z-score. Homozygous deletion of CDKN2A is demonstrated by near-zero expression of this gene across all exons. Tumor EWS125 and cell lines 6647 and CHLA-9 have focal loss of expression of a single exon. (PDF) [file pgen.1004475.s007.pdf]

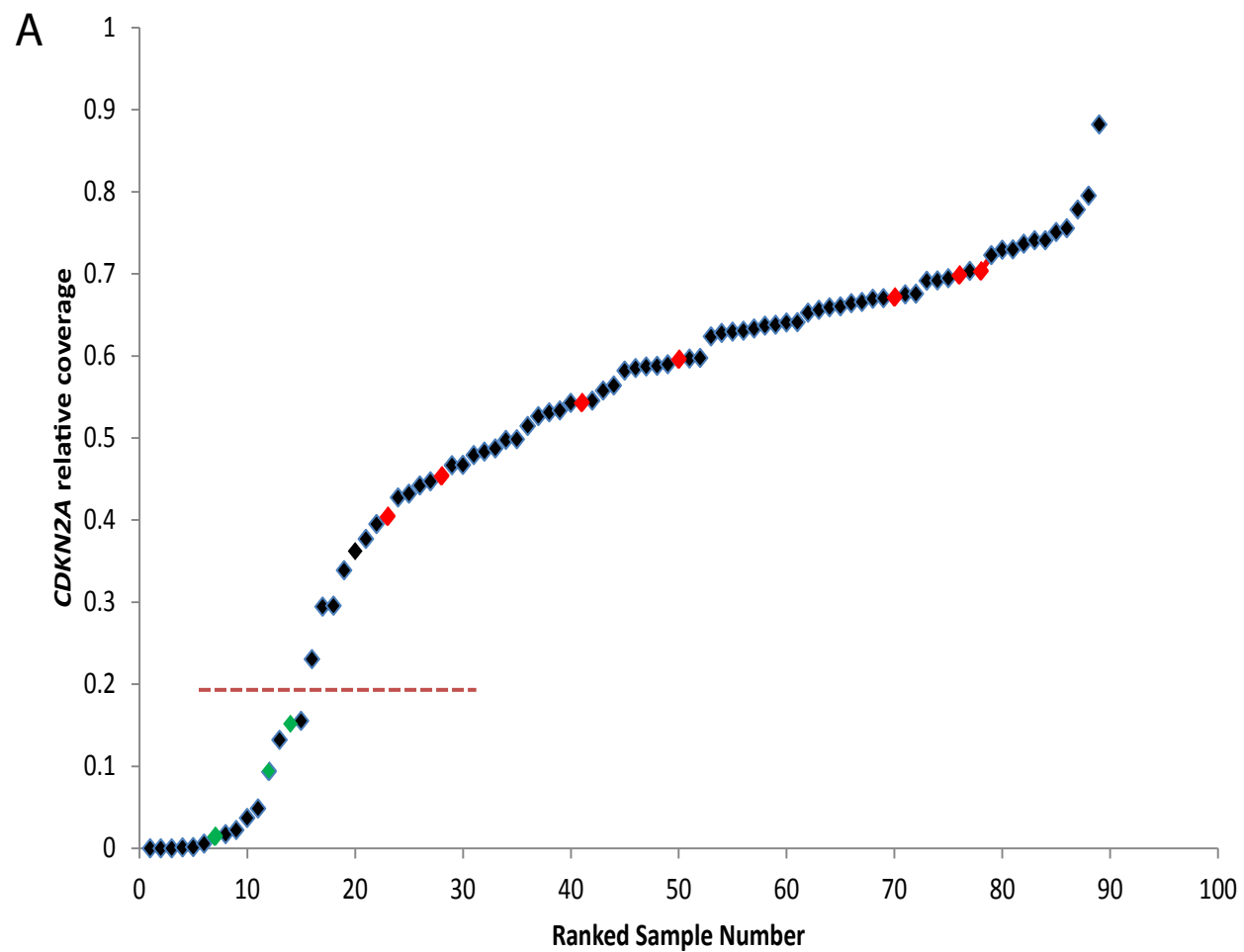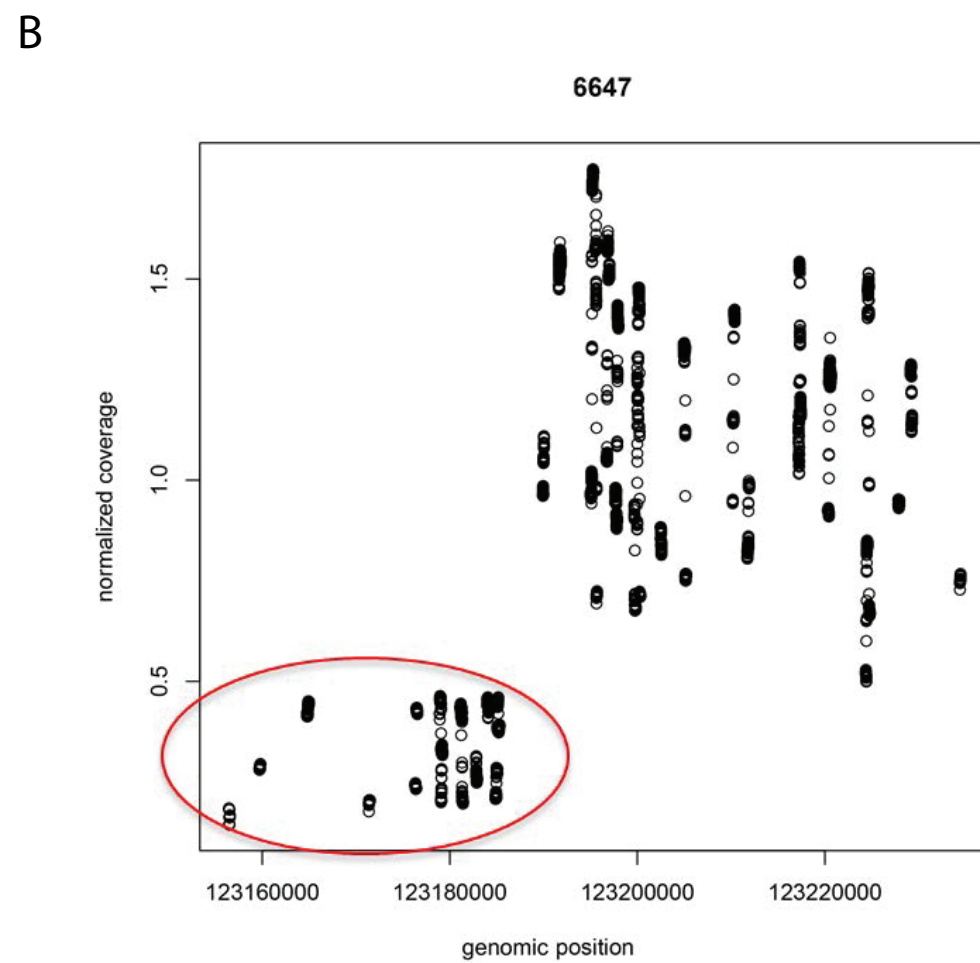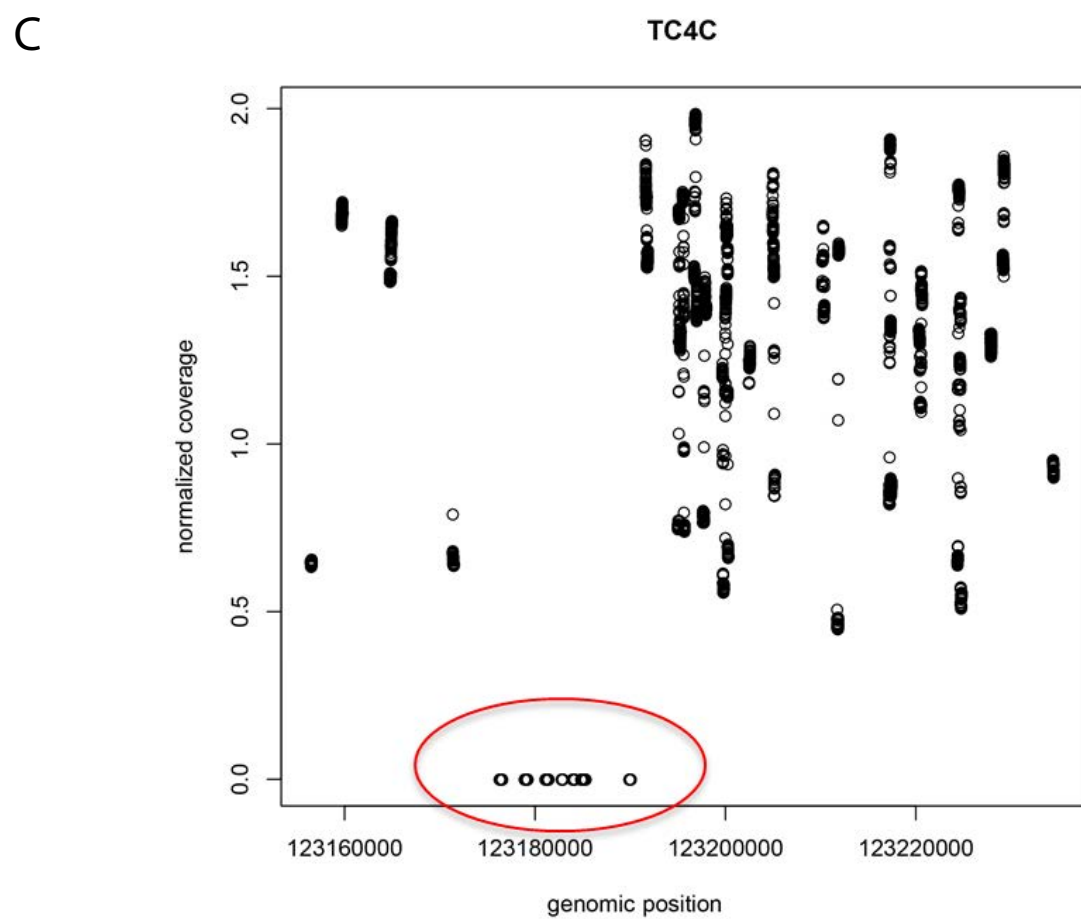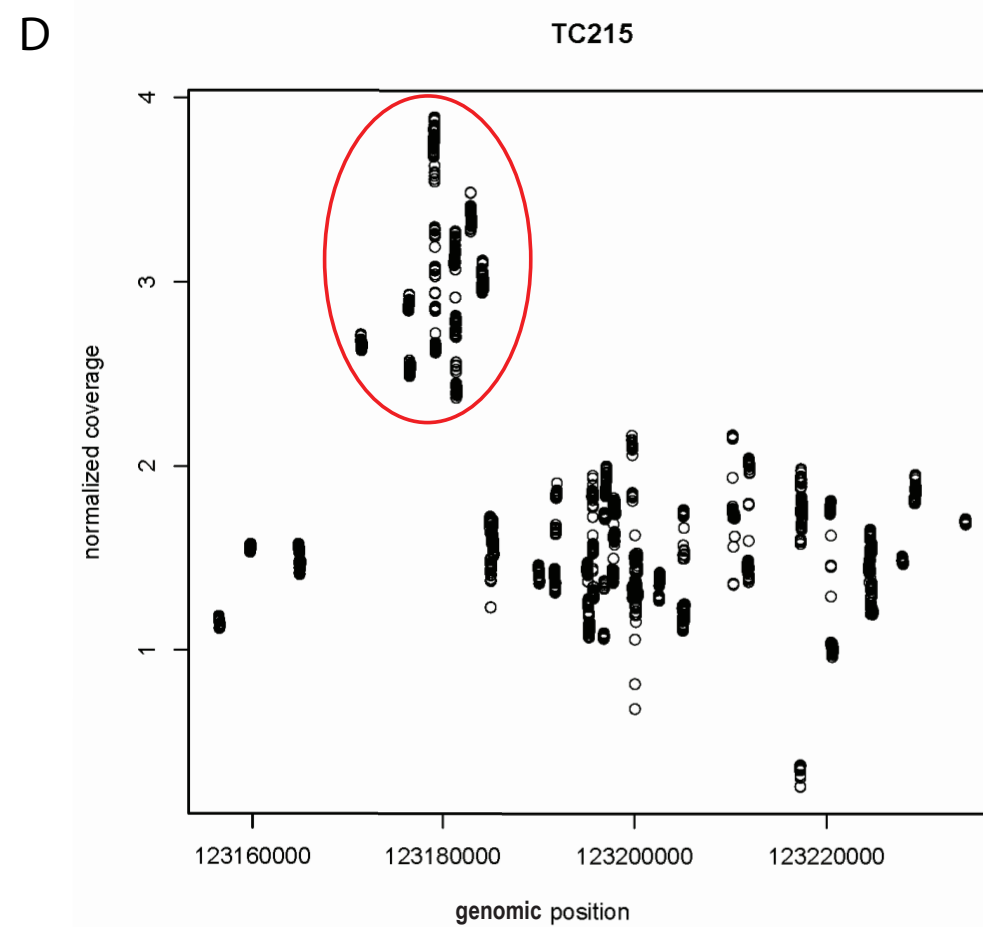

Supplement: Figure S8 — DNA sequencing coverage from the targeted sequencing approach was used to detect copy number alterations in recurrently mutated genes. A) Coverage in CDKN2A relative to average sequencing coverage in the same sample shows outlier samples predicted to have homozygous deletion of the gene. In a subset of samples, copy number status was assessed by SNP array to verify that CDKN2A was correctly predicted as deleted (green) or wild type (red). B–D) Copy number alterations seen in STAG2 based on normalized sequencing coverage plotted against genomic position on the X chromosome. B) Cell line 6647 contains a heterozygous deletion containing the 1st–11th coding exons of STAG2. C) Cell line TC-4C has a hemizygous deletion in the 5th–12th coding exons of STAG2. D) Cell line TC-215 has an intragenic duplication event as evidenced by doubling of copy number across contiguous exons. (PDF) [file pgen.1004475.s008.pdf]

A

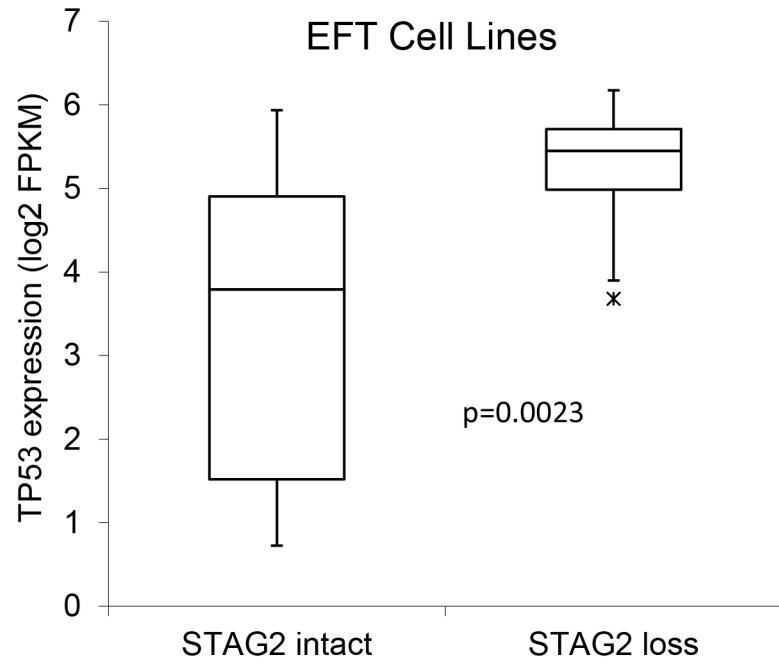

B

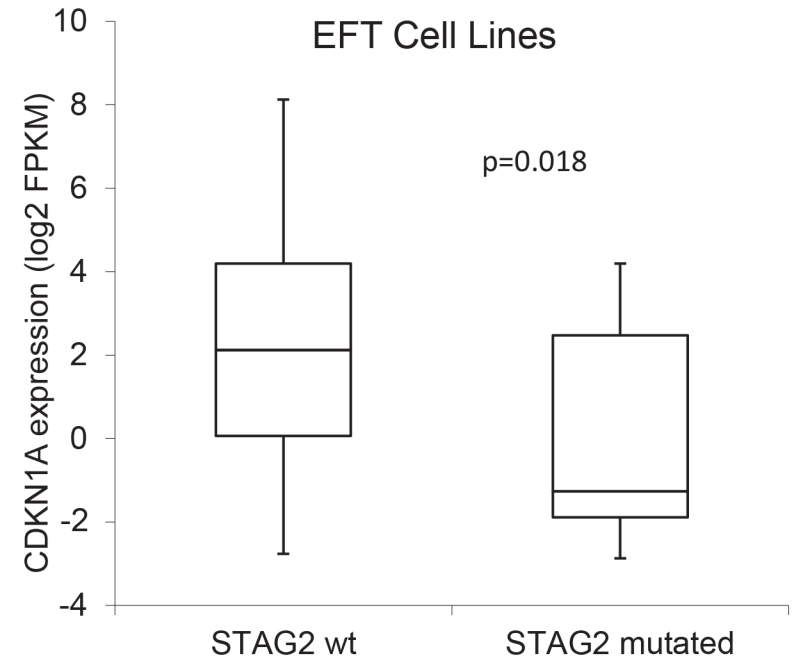

C

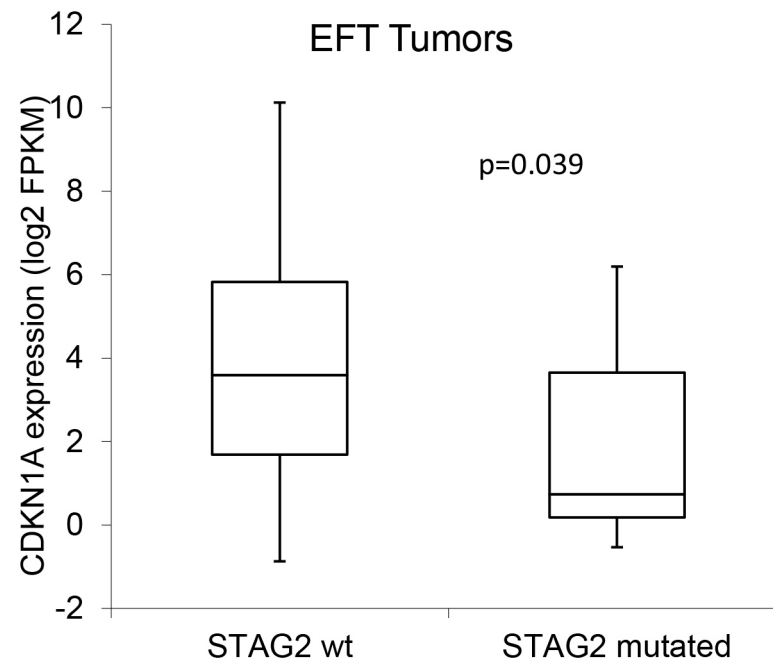

Supplement: Figure S9 — Box and whisker plots showing range and percentiles of RNA expression (log2 FPKM) in subgroups determined by STAG2 status. A) EFT cell lines with STAG2 loss have increased TP53 expression. B–C) EFT cell lines (B) and tumors (C) with STAG2 mutation have decreased CDKN1A expression. (PDF) [file pgen.1004475.s009.pdf]

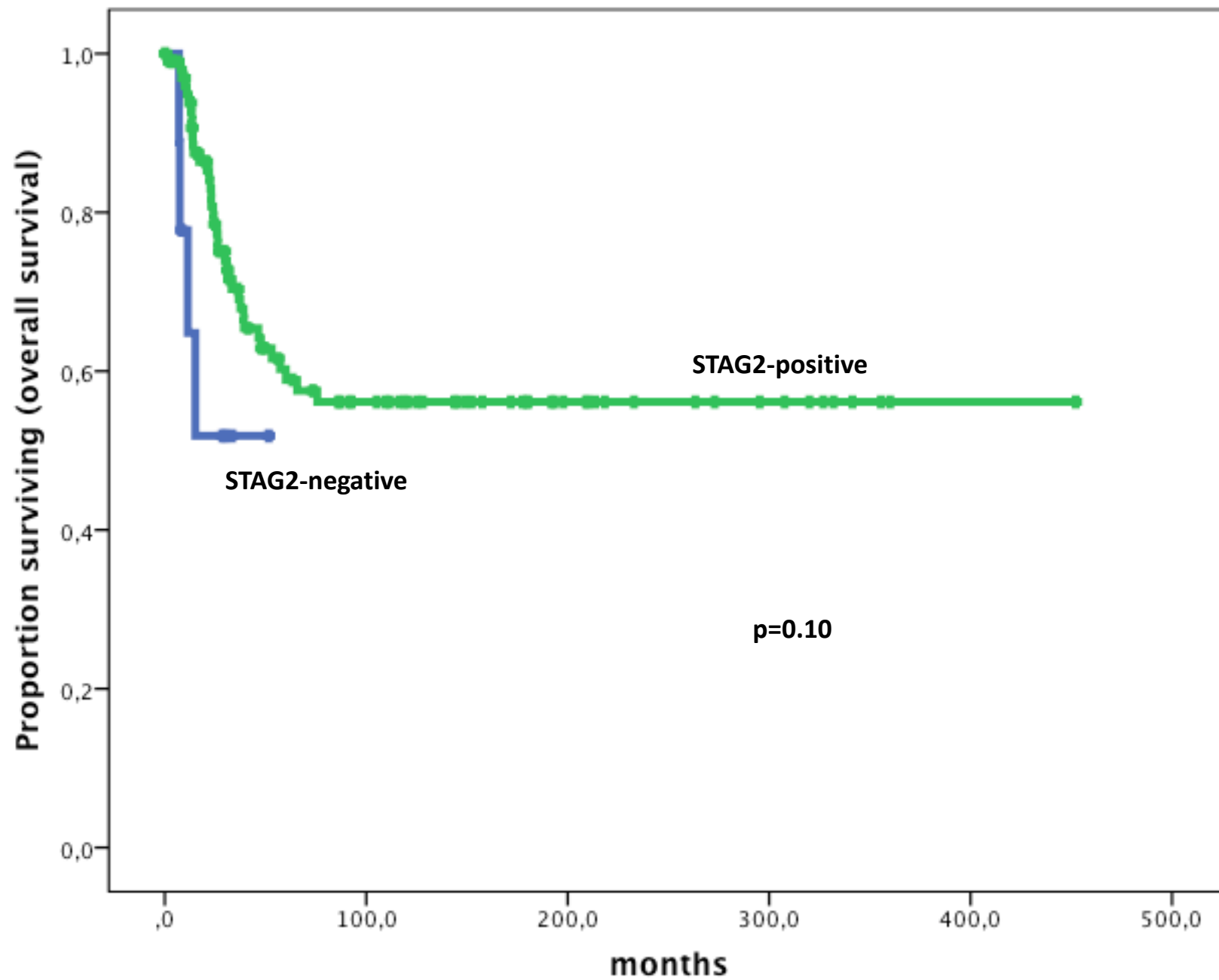

Supplement: Figure S10 — Cumulative overall survival of patients with primary, non-metastatic tumors in the TMA cohort stratified by STAG2 IHC status. This analysis demonstrates a trend towards decreased survival in patients whose tumors have loss of STAG2 expression. (PDF) [file pgen.1004475.s010.pdf]
