# Supplementary material for: The Genomic Landscape of the Ewing Sarcoma Family of Tumors Reveals Recurrent STAG2 Mutation
Source: PLoS Genet. 2014 Jul 10;10(7):e1004475. doi: 10.1371/journal.pgen.1004475 (PMC4091782; doi:10.1371/journal.pgen.1004475)
Supplement: Figure S5 — STAG2 immunohistochemistry in Ewing sarcoma tissue microarrays. STAG2 is robustly expressed in the majority of samples (left) but expression is completely lost in a subset of tumors (right). In STAG2 negative samples, expression is retained within the non-neoplastic stromal and endothelial cells, demonstrating the somatic nature of STAG2 loss in these tumors. (PDF) [file pgen.1004475.s005.pdf]

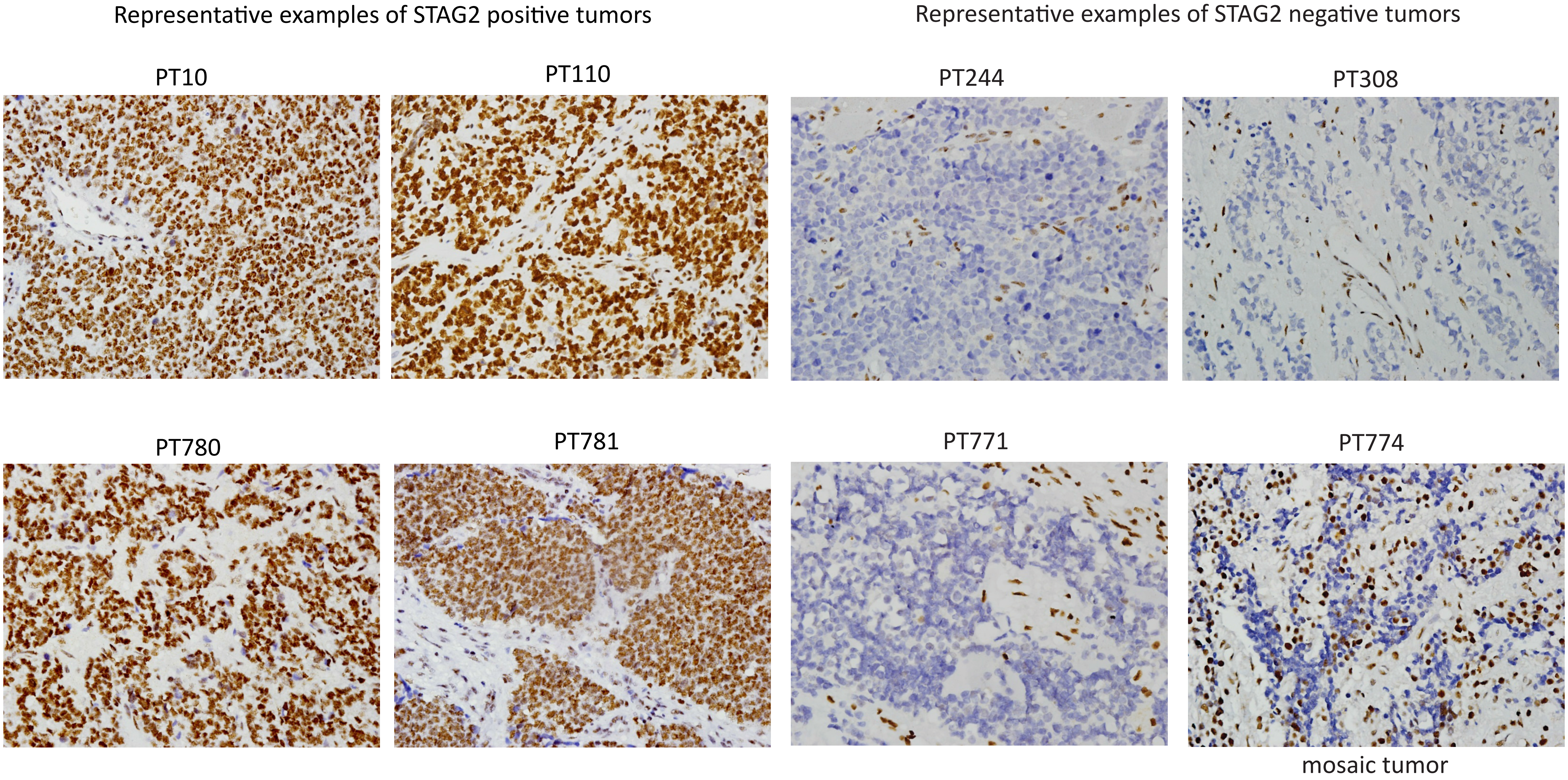

Representative examples of STAG2 positive tumors
Representative examples of STAG2 negative tumors
PT10
PT110
PT244
PT308
PT780
PT781
PT771
PT774
mosaic tumor
