# Supplementary material for: The Genomic Landscape of the Ewing Sarcoma Family of Tumors Reveals Recurrent STAG2 Mutation
Source: PLoS Genet. 2014 Jul 10;10(7):e1004475. doi: 10.1371/journal.pgen.1004475 (PMC4091782; doi:10.1371/journal.pgen.1004475)
Supplement: Text S1 — Clinical course and pathological description of tumors that harbor an alternate fusion. (DOCX) [file pgen.1004475.s022.docx]

**Supplementary Text 1:**

The patient whose tumor was found to have the *ETV6-NTRK3* fusion was diagnosed at age 7 months with a pelvic sarcoma without evidence of metastasis. He was originally treated as per protocol AEWS0031 (vincristine, doxorubicin, cyclophosphamide alternating with ifosfamide and etoposide), including surgery and radiation to the pelvis. Five years after diagnosis the patient had a pulmonary relapse. Salvage chemotherapy was administered as per protocol AEWS0521 (cyclophosphamide, topotecan, vincristine and bevacizumab). The patient also underwent consolidation with busulfan and melphalan, followed by an autologous stem cell transplant rescue. The patient had a second pulmonary relapse 10 years after initial diagnosis, and a right pulmonary nodule was resected. Pathology from the resected lung nodule showed polygonal and spindle cells noted to be atypical in morphology for EFT. The tumor cells were strongly positive by immunohistochemistry for CD99 and bcl-2 and negative for keratins AE1/AE3/CAM5.2, EMA, S-100, SMA and myogenin.

The patient whose tumor was found to have a novel *CIC-FOXO4* fusion was diagnosed at age 13. He initially presented with a peanut-sized, non-tender, non-erythematous mass on his posterior scalp. Resection was scheduled for 4 weeks after initial presentation, by which time the mass had grown to be egg-sized. Pathology revealed a small round blue cell tumor that stained positive for CD99 and negative for desmin, HMB45, keratins AE1/AE3, and S-100. Clinical testing was negative for *EWSR1-FLI1* fusion. The patient received treatment as per protocol AEWS0031 (vincristine, doxorubicin, cyclophosphamide alternating with ifosfamide and etoposide). Five months after diagnosis, the patient had an excision of the surgical scar, which was negative for tumor. He relapsed 11 months after his initial diagnosis with 3 scalp nodules, two of which were resected and were small round blue cell tumors with identical immunohistochemical profile as his initial tumor. Unlike in the primary tumor, the recurrence specimen showed pseudorosettes such as that is typical for primitive neuroectodermal tumors. Shortly thereafter, the patient had a restaging chest CT scan that revealed several pulmonary metastases. The patient received salvage chemotherapy with irinotecan and temozolomide and then cabozantanib, the latter on clinical trial. The patient passed away 22 months after his initial presentation.

The novel *FUS-NCATc2* fusion was found in the tumor of a male diagnosed at age 15 with Ewing sarcoma of the right femur. The pathology report noted multiple areas of necrosis with the majority of tumor cells consistent with a small round blue cell tumor. In focal areas there was a distinct spindle appearance, and other foci had chondroid differentiation. No additional pathological or clinical history was available for this patient.
